# Congress Abstracts

**DOI:** 10.1155/S1463924682000261

**Published:** 1982


Journal of Automatic Chemistry, Volume 4, Number 2 (April–June 1982), pages 90–106

### International Congress on Automation in the Clinical Laboratory: Editorial

The last number of *Journal of Automatic Chemistry* marked the first edition produced by our new publisher, likewise this second issue also marks a new departure. Part of this issue is devoted to the publication of abstracts for the International Congress on Automation in the Clinical Laboratory. This meeting is being held in Barcelona from 19 to 22 April 1982, and the abstracts are devoted to various aspects of automation as applied to the clinical laboratory. However, the problems of applying computers to instrumentation, or to the general work-flow in any laboratory, present similar problems to the user and to the systems designer and there is considerable merit in sharing experiences. Further details on the papers abstracted can be obtained directly from their authors, although I hope that several of them will submit the full text of their papers for publication in the *Journal*. Every participant at the congress will be presented with a copy of the *Journal* with their registration documents. If it is the first time that they have seen the *Journal*, we welcome them as readers and hope that within these pages they will find much of value to their work. I also hope that they have a valuable and enjoyable congress and gain much of value from their participation.

On a personal note, I have just returned from a brief visit to the 33rd Annual Pittsburgh Conference and the large exhibition will be presented in the next issue. There, during a discussion with two members of my main editorial board—Professor Howard Malmstadt and Dr Rolf Arndt—about inviting Professor G. Horlick to the editorial team, a very interesting suggestion was raised. It was proposed that the formation of a society of laboratory automation would be an attractive concept and that it should be directly linked to the *Journal*. This should encourage a broader subscription base for the *Journal* and promote conferences on laboratory automation where clinical chemists and industrial chemists, along with those in academic life, can discuss the many problems involved in introducing computerization and automation into various work environments. The proposal certainly has considerable appeal and I would welcome reactions from our readers. A world-wide conference on laboratory automation, along the lines of the congress in Barcelona, would be a valuable forum for discussing the problems and advantages of automation.

An interesting development at Pittsburgh was the introduction of an automated system based on robotics. The Zymark Corporation was formed in March 81 to develop, manufacture and market instrumentation for automated laboratory-scale processes used in chemistry and biochemistry. The Zymate Laboratory Automation system combines robotics and laboratory stations to automate the procedures used in sample preparation. For example liquid handling, sample conditioning, separation and chemical modification. In addition to the instrumentation, the Zymark Corporation has developed an educational program to provide a foundation in modern techniques of sample preparation—this program is available on a subscription basis. Whilst the approach is not totally new—the Robot Chemist and similar systems were launched more than 10 years ago—the technology now available makes the chances of success of this new venture more likely. Future developments along similar lines are eagerly awaited.

**Peter B. Stockwell**

### Congress Abstracts

### Session 20.1: Clinical Chemistry—General

### 20.1.1: A colorimetric alpha-amylase method on automated analysers

*By J. L. Derocque, Boehringer Mannheim GmbH, Mannheim, FR Germany*

A survey of the various methods for the assay of alpha-amylase activity in serum and urine is presented. Their suitability for application to automated instruments is discussed, and a new kinetic alpha-amylase method using para-nitrophenyl-maltoheptaoside as a chromogenic substrate is described. This method is free from interference from endogenous glucose and can easily be adapted to most of the common automated systems.

An international inter-laboratory survey including more than 1000 laboratories in 14 European countries was carried out. The participants in this survey were requested to report simultaneously the results they obtained with the new method and the results of the method routinely used in their laboratory. Evaluation of the results showed that the inter-laboratory coefficient of variation (CV = 12%), obtained with the new method was good and comparable with those obtained for other enzymes (for example alkaline phosphatase, gamma-GT) in surveys carried out in Germany. The inter-laboratory precision was also far better than that obtained with most of other alpha-amylase methods.

Results are also discussed according to the various instruments used in this survey.

### 20.1.2: Determination of serum urea with use of o-phthaldehyde reagent in the Coulter chemistry CA-3 and ABA-100

*By J. M. Paz, A. Lopez-Urrutia, J. C. Tutor and Del Rio, Laboratorio Central, Hospital General de Galicia, Universidad de Santiago de Compostela, Spain*

The o-phthaldehyde procedure, which was developed by Jung for the measurement of urea, was adapted to the Coulter chemistry CA-3 and ABA-100 and evaluated for accuracy, precision, linearity and sample-to-sample interaction. The results obtained were compared with those for the urease/GLDH and diacetyl methods.

The precision obtained was good (CP $>98{\cdot}5$) and the total analytical error was not medically significant. The linearity throughout was 1·33–44·96 mmol/l. A sample-to-sample interaction was observed only for Coulter chemistry. For 121 random samples diacetyl (Coulter Chemistry) versus o-phthaldehyde (Coulter chemistry) gave a Pearson coefficient ($r$) of 0·998 ($y = 0{\cdot}98 + 0{\cdot}10$). For 63 samples o-phthaldehyde (Coulter chemistry) versus urease/GLDH (ABA-100), $r = 0{\cdot}998$ ($y = 0{\cdot}97x + 0{\cdot}18$). For 75 samples diacetyl (Coulter chemistry) versus o-phthaldehyde (ABA-100), $r = 0{\cdot}999$ ($y = 0{\cdot}99x + 0{\cdot}13$).

The Jung procedure can be simply adapted to the Coulter chemistry and ABA-100. It provides an accurate, precise and economical alternative to the diacetyl and urease/GLDH methods.

**20.1.3: An automated procedure of serum iron on the Technicon AutoAnalyzer II**

*By Jau-Wen Chu and Clara V. Sumeghy, Henry Ford Hospital, Detroit, Michigan 48202, USA*

Comparison of Technicon AutoAnalyzer II method of serum iron (method No. 25, March 1972) to that of AutoAnalyzer I (method N-62P) yields an average of 5% higher in serum iron. The spuriously high result of the former is due to the positive Donnan effect resulting from the imbalance of ionic species between sample and recipient streams. This can be corrected by reformulating the reagents as follows: iron acid reagent, 5% NaCl and 0·01% Tween 20 in 0·11 N HCl; acid diluent, 1% ascorbic acid in the iron acid reagent; iron colour reagent, 0·035% ferrozine and 0·01% Tween 20 in 0·11 N HCl; and sodium acetate, 1·0 N. The flow diagrams remained unchanged. Lag phase and half-wash time, studied by the decay of steady-state curve, were found to be 4·1 s and 5 s, respectively.

The modified method compares well with AutoAnalyzer I, with a correlation coefficient of 0·995, a slope of 1·05, and an intercept of −3·7 μg/dl. The recovery rate, studied by the addition of aqueous iron solution to patient sera, was found to average 98·2%. The day-to-day coefficient of variation during the two months' study was 3·6%. The method is not affected by the normal concentrations of copper present in the sera. Thioglycollic acid, which chelates to serum copper, can replace ascorbic acid in the acid diluent and provides a longer reagent shelf-life. This automated procedure for serum iron has been used in the authors' laboratory for over three years, and has proved to be a simple and reliable method.

**20.1.4: An improved creatinine procedure of high specificity for the Cobas-Bio Analyser**

*By M. Sheehan and J. Salmon, Good Samaritan Hospital, Portland, Oregon 97210, USA*

The authors have adapted the end-point procedure of Slot (see *Scandinavian Journal of Clinical Laboratory Investigation*, **17** [1965]) to the Cobas-Bio centrifugal analyser. The two-reagent addition capability of the instrument allows complete automation of this manual procedure. Sample and alkaline picrate reagent are added to the reaction cuvette and allowed to react for 5 min. After an absorbance reading is taken, an acidic reagent is added and another absorbance reading is taken 60 s later. The second absorbance reading (colour due to interfering substances) is subtracted from the first (colour due to picrate complexes plus interferences), and the analyser calculates creatinine concentration of samples based on this difference relative to standards. Within-day ($N = 20$) and day-to-day ($N = 10$) precision studies exhibited percentage CVs of less than 1 and 1·7, respectively, at the 6·3 mg/dl level. The method is linear to at least 15 mg/dl and exhibits good correlation with the ASTRA-8 kinetic alkaline picrate method. No interference was seen for samples from diabetics in keto acidosis.

**20.1.5: Pyridoxal-5-phosphate aminotransferase method for Autoanalyser II Y SMA 12/60**

*By J. Perez Garcia-Buela, Servicio Analisis Clinicos, Ciudad Sanitaria Juan Canalejo, La Coruña, Spain*

A pyridoxal-5-phosphate (P5P) activated aminotransferase (AST) method for use on Technicon's continuous-flow equipment is described (the AA II and SMA 12/60). The method is in line with the recommendations of the International Federation of Clinical Chemistry (IFCC). A substrate, according to the IFCC's method, with 30 mM pyridoxal-5-phosphate was used. No pre-incubation of the sample with P5P or manifold changes are required. With this procedure the linearity, at 37°C, was to 500 U/l. The following results were obtained:

| | Level 1 | Level 2 |
|---|---|---|
| Number of assays | 51 | 54 |
| Media (U/l 37°C) | 63 | 215 |
| SD (U/l) | 2·24 | 1·93 |
| CV (%) | 3·55 | 0·89 |

Samples from a normal donor group and from a group of patients with chronic liver disease were studied with the AST method. The normal donor group ($N = 42$) showed an increase of AST to 7–10 U/l with added P5P. Patients in the chronic liver disease group who had near-normal AST values showed the same increase as the normal group, while the patients with elevated AST values showed increases as high as 100%.

**20.1.6: Automated calculation of gamma glutamyl transpeptidase in alcoholics**

*By P. Bataller Martinez and V. Sanchis-Bayarri, Clinical Analysis Service, Provincial Hospital, Valencia, Spain*

It is sometimes difficult to evaluate the effects of alcohol on the hepatic cell. Patients have a tendency to minimize their consumption of alcohol in their clinical histories. One of the objective methods is to calculate quantitively the gamma glytamyl transpeptidase (GGTP). The results of analyses of GGTP in patients from various hospital centres were studied over a three-month period. An auto-analyser (ABA-100) with a 450 filter was used, samples were incubated at 37°C, sample volume was 5 μl and the analysis time was 5 min. The results showed an increase in the GGTP in patients with a clinical history of presumed alcoholism, even in the absence of a rise in other enzymes, such as serum glutamate-oxalacetate (SGOT), serum glutamate-pyruvate (SGPT) and alcaline phosphatase (AP). In some patients, where the aim is to reduce the intake of alcohol rather than total abstinence, the results of a series of measurements of GGPT provides an objective control of alcohol consumption.

**20.1.7: Automated determination of serum β-N-acetyl-D-glucosaminidase**

*By G. Johannsen, D. Maruhn and D. Paar, Division of Clinical Chemistry, Department of Medicine, University of Essen, FR Germany*

Pott *et al.*'s discontinuous manual assay for serum β-N-acetyl-D-glucosaminidase (EC 3.2.1.30, AGS) was adapted to the ACP 5040 Eppendorf discrete automated analyser. The instrument was run in end-point mode at a throughput rate of 120 samples/h. The analyser was equipped with a thermojacketed flow-through device to provide the dispensers with pre-heated (at 40°C) reagents. In the assay, 25 μl of sample was added to 250 μl of buffer-substrate solution (citrate buffer, 0·05 mol/l, pH 4·2; 4-nitrophenyl-β-N-acetyl-D-glucosaminide, final concentration 9 mmol/l). After 6·5 min. incubation at 37°C the reaction was terminated by the addition of 250 μl of 2-amino-2-methyl-propan-1-ol/HCl (AMP) buffer at pH 10·25. Absorbance of liberated 4-nitrophenol was read against a blank to which AMP buffer was added before incubation.

A linear relationship was found between the amount of enzyme in the assay mixture and enzyme acitivity up to 100 U/l. Within-run imprecision (CV) ranged from 0·89 to 4·78%. Between-day imprecision, as determined with Hyland N 11 accuracy control serum, amounted to 4·09% ($N = 9$, CV). Comparison with the manual method yielded a correlation coefficient of 0·9885. The automated ACP 5040 procedure is fast, precise and reliable for the determination of AGS in human serum.

**20.1.8: Determination of non-pregnancy total urinary oestrogens by fluorescence photometry: comparison between a classical extraction and an automated Kober-Ittrich method**

*By R. Wolfrum, M. Trapp, H. G. Bohnet, and F. Leidenberger, Chemistry Department, Allgemeines Krankenhaus Heidberg and Institute for Hormone and Fertility Disorders, Hamburg, FR Germany*

Halket and Wolfrum published a paper in 1980 which showed that a significant correlation exists between a manual and an automated continuous flow technique for 24 h urinary total oestrogen determination in pregnancy (mg range). The present study is a further contribution to the development of a reference method and describes the analytical results in 93 urine samples, in the concentration range 6–200 $\mu$g/24 h, using the same automated system as the previous project (Breda Scientific) with a preceding Sephadex G-10 step (van Kessel) and Brown's semi-automatic method. Regression analysis of the paired values shows a linear relationship, $y = 1{\cdot}14x - 3{\cdot}36$ ($y$ = flow technique), and a correlation coefficient ($r$) of 0·97 ($p < 0{\cdot}001$). At present the automated flow technique is the only system for the simultaneous determination of dU-mg and $-\mu$g concentrations. Its use should allow external quality-control procedures to be implemented.

**20.1.9: Chamber analytical technique—its application to enzymatic and immunological assays in the ultra micro range**

*By E. Hoffmann-Blume and A. Horn, Institute of Physiological Chemistry, Friedrich-Schiller University, Jena, German Democratic Republic*

The chamber analytical technique allows the performance of colorimetric and fluorimetric tests in the form of fixed-point or kinetic measurements in sets of 50 samples with test volumes between 8 and 80 $\mu$l at light paths between 0·2 and 2 mm.

A new device is described for temperature-controlled automated fluorimetric and colorimetric measurements with increased sensitivity by directing the light beam twice through the solution for colorimetry. Precisions are in the range of $5\text{–}8 \cdot 10^{-4}$ units of absorbance and 0·5% for fluorimetric measurements. The system is accomplished by automated volume dispensing and accessories for pre-analytical steps.

Application examples are reported: determinations of alanine aminotransferase activities as well as of $K_M$ and $V_{max}$ of alkaline phosphatase in 80 $\mu$l tests result in day-to-day CVs of 2–5% and 6–7%, respectively. An ultra micro ELISA for $\alpha$-fetoptotein in serum with test volumes of 10 $\mu$l and a CV of < 10% demonstrates the applicability for fluorimetry. About 150 assays can be carried out with 1 ml of antiserum for turbidimetric determinations of albumin and apoprotein B (inter-assay CV 4–8%). With the measuring and volume dispensing automation 100–200 miniaturized tests/h can be performed.

**20.1.10: An emergency laboratory integrated within a laboratory data processing system**

*By D. Neumeier, H. Sator, G. Rindfleisch and M. Knedel, Institut für Klinische Chemie, Klinikum Großhadern, Ludwig-Maximilians-Universität München, D-8000 München 70, FR Germany*

The data-processing system for the emergency laboratory was integrated in the authors' clinical laboratory computer system (PRIMULAB) with its prime objective towards the service requirements of the laboratory. Therefore it includes possibilities of simultaneous optical reading of request forms and on-line capturing, processing and printing of laboratory test data. Priority request forms, which allow the clinician to indicate the interval after which emergency test results should be available, are registered by an optical reader and arranged according to urgency by the computer. Work-sheets are replaced by displaying all the information required for accurate specimen analyses on a large, colour TV screen. The individual processing status of all tests, from as many as 30 request forms, is displayed in a colour code. For process control the updated delay time for test performance is faded in.

The computer capability for managing the sample throughout and data processing minimizes the stress on technicians. This results in a reduction of the turnaround time of tests. 95% of all requested tests are performed and reported within the interval indicated. In critical situations, test results are available within 3–10 min.

**20.1.11: Experiences in a clinical laboratory gained by a Hungarian-made programmable automatic chemical analyser**

*By L. Bartalits and B. Bak, Hospital in Péterfy S, str. 8, Budapest 1076, Hungary and Central Research Institute for Physics, Budapest, Hungary*

The necessary conditions for the automatic instrumentation of enzyme activity determinations are examined, the operational details of the CRIP clinical chemical analyser are introduced and 18 methods for measuring enzyme activity and metabolite-concentration of body fluids are presented. The analyser is in a discrete system operated by compressed air and has a throughput of 200 samples/h. The measurement of metabolites is performed at equilibrium point, the measurement of enzyme activity is performed by two-point kinetics, according to the Trayser-Saligson principle in an optimized reaction environment. The iron-free components of the analyser make measurement of iron by a photometric method possible.

### Session 20.2: Clinical Chemistry—Evaluation

**20.2.1: The evaluation of a high-speed (9000 tests/h) multichannel (30 channels) automated analyser for clinical chemistry**

*By Yasuyuki Hayashi and Masakazu Hineno, Department of Clinical Pathology, Juntendo University School of Medicine, 3-1-1 Hongo, Bunkyo-ku, Tokyo and R/D Engineering Department, Analytical Instruments Division, Shimadzu Corporation, Kyoto, Japan*

The Shimadzu Multichannel Analyser CL-30 was developed by the Shimadzu Corporation (Kyoto, Japan); Japanese professionals co-operated in the development—this co-operation is a guide-line of the Ministry of International Trade and Industry of Japan. The new analyser is intended to realize high capacity with minimized volumes of specimen and reagents, as well as to be supplied at a low price. It has a characteristic disposable sheet made from polyester resin, which is used for reaction vessels and cuvettes, and is termed a 'micro-cuvette-sheet'. The repertoire of this analyser includes serum enzymes, lipids, sugars, proteins, nitrogen compounds and other contents by kinetic assay, visible colorimetry or flame photometry. Evaluation data are discussed; the machine has been studied since May 1980. Within-day precision (as CV) was 0·5–3% and day-to-day precision was 2–8%, these precisions are acceptable when compared with the usual method in the authors' laboratory. Approximately 5% carry-over was found, which is probably due to the Teflon transportation tube and so may be improved on.

Analytical programmes are changeable by altering the position of a photometer or by adding optional accessories. The Shimadzu CL-30 is capable of performing fast analyses with small specimen volumes and reagent consumption.

**20.2.2: Evaluation of a new centrifugal analyser: Flexigem, developed by Electro-Nucleonics Inc.**

*By Y. Gourmelin, J. Herfent, G. Glikmanaf and A. Truchaud, Department of Biochemistry, Meaux Hospital, France*

The instrument was evaluated for two months in the authors' laboratory. (1) Precision at three different levels of urea was determined over a period of 20 days, using a kinetic urea reagent. Within-run CV was better than 2·5% and day-to-day precision was better than 4%. (2) Contamination was tested by means of a kinetic Jaffé method for creatinine. (3) Temperature accuracy and stability were tested at 25, 30 and 37°C, using a cresol-red solution. (4) The linearity and accuracy of the optics were tested at 340, 405 and 500 nm. (5) Special attention was given to the micro-computer allowing the user to choose between four curve-fit calculation modes: 4 parameter log-logit, 5 parameter logit, 5 parameter exponential, 5 parameter polynomial. A quantitative turbidimetric IGG method was used to test the polynomial mode. The 5 parameter logit mode was tested by means of EMIT gentamicin. In both cases the program proved to operate satisfactorily.

### 20.2.3: Application of the Flexigem analyser for routine laboratory analysis

*By E. Knoll, K. Dettmer and H. Wisser, Robert-Bosch-Krankenhaus, Department of Clinical Chemistry, Auerbachstrasse 110, 7000 Stuttgart 50, FR Germany*

The authors have eight years' experience with a centrifugal analyser of the first generation—Gemsaec. They investigated the performance of the newly developed Flexigem, a centrifugal analyser of the third generation, and compared it with Gemsaec. It was tested over a period of three months, its practicability, accuracy and precision were examined. Three enzyme determinations (GOT, GPT and Gamma-GT), four substrate determinations (glucose, creatinine, triglycerides and urea) as well as the turbidimetric determinations of IGG, IGA and IGM were tested. Precision was established using control sera. Accuracy was determined by comparing analyses of patient samples, covering as wide a concentration range as possible. The authors report a good correlation between the Flexigem methods and those of Gemsaec.

### 20.2.4: Investigations of the activity of some enzymes by microcentrifugal analyser: IL Multistat III

*By J. Jordanova, Higher Medical Institute, Central Clinical Laboratory, Sofia, Bulgaria*

The activity of aspartat aminotransferase (AsAT), alanin aminotransferase (AlAT), lactate dehydrogenase (LDH), α-hydroxibutira dehydrogenase (HBDH) and creatine phosphokinase (CPK) in serum was studied with the microcentrifugal analyser IL Multistat III programmed for Boerhinger's tests. The possibilites of the Multistat III increasing the number of the samples and decreasing assay time was investigated. Multistat III is shown to reduce the volume of the biological material necessary, as well as the volume of the reagents. The within-day and day-to-day reproducibility and accuracy ($N=20$) were examined using five commercial control sera.

### 20.2.5: Evaluation of the Hitachi 705 automatic analyser

*By C. Ferre, M. J. Castiñeiras, M. J. Alsina, J. Riera and R. Galimany, Departmento de Analisis Clinicos (C.S.), 'Principes de España', Hospitalet de Llobregat, Barcelona, Spain*

The Hitachi 705 automatic analyser was tested against the standard procedure laid down by the Instrument Commission of the 'Sociedad Española de Quimica Clinica'. The parameters investigated for the evaluation were: (1) linear ranges; (2) within-run and inter-run imprecision, using three different concentration levels; (3) contamination effects.

The serum constituents used in the trials were: glucose (GOD-PAP), urea (urease UV), creatinine (Jaffé without deproteinization), calcium (cresolphtalein), AST (optimized standard method) and CK (optimized standard method, NAC activated). In addition the Hitachi 705 was assessed for inaccuracy against the Technicon SMAC.

### 20.2.6: Serum urate determinations on the Hitachi ABCA 706 D

*By L. Lepoutre and S. Pauwels, U.I.A., Academic Hospital, Wilrijkstraat, 10 B2520 Edegem, Belgium*

Serum urate determinations using a chromogen-based method were performed on the Hitachi 706 D and compared with an end-point analysis on the CentrifiChem 400. Analyses were performed at 25°C on the Hitachi and at 30°C on the centrifugal analyser. Precision and linearity were studied on appropriate dilutions prepared from pooled serum, saturated with uric acid, while calibration was based on external quality-control data. The following results were obtained: within-run precision: 0·13 mmol, $N=30$, CV = 1·47%; 0·25 mmol, $N=31$, CV = 2·08%; 0·47 mmol, $N=30$, CV = 0·69%; 0·88 mmol, $N=24$, CV = 0·99%. Between-run precision: ($N$ = consecutive days) 0·26 mmol, $N=30$, CV = 2·1%; 0·62 mmol, $N=30$, CV = 2·0%. Linearity was found to exceed 1·98 mmol. Correlation between the two methods can be represented by $y = 1{\cdot}090\,x - 0{\cdot}656$ with $r = 0{\cdot}978$ on 163 serum samples (where $y$ = chromogen method, $x$ = centrifugal technique). Carry-over was observed from concentrations higher than 0·82 mmol.

### 20.2.7: Evaluation of chemistry and Emit assays on Flexigem, a new centrifugal analyser

*By F. Van Lente and V. M. Leitz, Electro-Nucleonics Inc., 368 Passaic Ave., Fairfield, New Jersey 07006, USA*

Flexigem is a microprocessor-controlled centrifugal analyser, which in addition to performing routine chemistry procedures, can be programmed to perform immunoassay and special chemistry procedures. Data for non-linear immuno-assays can be fitted to generate standard curves using one of four mathematical models: log-logit, four parameter polynomial, exponential, and five parameter logit. Thirty such curves can be stored in memory at any one time. The performance of 30 routine chemistry procedures, four Emit procedures and three immunoglobulin procedures has been evaluated using protocols based on NCCLS PSEPs 2, 3, and 4. Precision data obtained at three levels for each analyte and method comparison data are presented. Precision data for key assays is:

| Within-run imprecision | $\bar{X}$ | % CV |
|---|---|---|
| Glucose | 242 | 1·2 |
| Phos. | 5·9 | 1·7 |
| Ur. Acid | 7·1 | 1·5 |
| Alk. Phos. | 204 | 2·3 |
| IgG | 896 | 4·5 |
| Phenobarb. | 35 | 3·4 |

| Total assay imprecision | $\bar{X}$ | % CV |
|---|---|---|
| Glucose | 242 | 1·5 |
| Phos. | 5·9 | 2·3 |
| Ur. Acid | 7·1 | 2·1 |
| Alk. Phos. | 204 | 2·8 |
| IgG | 896 | 9·4 |
| Phenobarb. | 35 | 5·1 |

Comparison studies have been performed at three sites for key assays. Data on Theophylline was essentially identical at the two sites: site 1, $r=0{\cdot}97$, $x=18{\cdot}2$, $\bar{y}=18{\cdot}1$; site 2, $r=0{\cdot}97$, $x=12{\cdot}8$, $y=13{\cdot}2$, and precision was statistically equivalent.

In conclusion, the Flexigem analyser has been shown to give precise results and values obtained have been shown to be in good agreement with those obtained using established procedures.

### 20.2.8: Experience with multichannel analysers in 26 Swedish hospitals

*By Kas Levin and Torsenten Aronsson, Clinical Chemistry Central Laboratory, Central Hospital, S 721 89 Vasterås and Clinical Chemical Central Laboratory, Academic Hospital, S 750 14 Uppsala, Sweden*

In October 1980 a workshop on multichannel analysers was arranged under the sponsorship of the Swedish Society for Clinical Chemistry. A mulitchannel analyser was defined as an instrument capable of carrying out more than 10 different analytical procedures on one sample, with a capacity of analysing at least 60 samples-standards-controls/h. The users of eight different systems were present at the meeting. The participants had been invited to analyse one common batch of a control serum in a standardized fashion before the meeting. The data were analysed using a statistical model, where the imprecision could be separated into within-day imprecision, between-day imprecision and total imprecision.

At the meeting the results were collected and discussed within the users' group, and the mean values for each type of multichannel analyser were calculated. The differences found between the performance of different analysers are small. These differences and the differences between different types of analysers are discussed by the authors.

### 20.2.9: Aurora, a new clinical analyser

*By Lars-Ake Carlsson, Clinicon AB, Box 148, S 161 26 Bromma, Sweden*

A novel instrument for kinetic and end-point measurements, the Aurora, is described. The instrument is a completely new development, having unique features in liquid handling and photometry. The Aurora analyser is able to selectively perform up to 16 analyses on a patient sample. It is equipped with a computer with a multi-task operating system making it possible to perform several tasks simultaneously, for example process control, input of requests, and printing of results. Sample and reagent handling is made by stepper-motor driven pumps. These pumps are highly precise (better than 0·5%) and accurate (better than 1%) and have negligible dead volume and very low carry-over (sample-sample 0·05–0·2%, method-method less than 1 ppm). Sample and reagent are incubated in tubes in a rotor and after 10 min. are sucked into a photometer for measurement. Three different reagent pumps and a special stepper mode of the reaction rotor makes it possible to add reagents twice at 12 different moments during an 11 min. period. The temperature of the two independent temperature control systems for the photometer and the incubation rotor are held within ±0·05°C from the stated temperature. The four-channel photometer is optimized for enzyme measurements (short path-length 2·5 mm) and shows low sensitivity to turbidity in patient samples and controls due to a unique optical system with a large acceptance angle. Calculation of the slope of zero-order reactions is made by linear regression, while first-order reactions are evaluated with an integration method. Rate curves are displayed on a VDU and are sorted on a discette if a quality figure is exceeded. Patient results are accepted only after passing an acceptance procedure, where for example, a VDU presentation of controls is made. From this picture drift any excessive variation during an analysis is easily seen. Results from an external evaluation of the instrument are presented.

### 20.2.10: Analytical experiences with Astra-8

*By A. Szabó, Postgraduate Medical School, Department of Clinical Chemistry, H1389 Budapest, P.O. Box 112, Hungary*

The Astra-8 analyser (Automated Stat-Routine Analyser, Beckman Company) with modules was used for assaying serum and urine sodium, potassium, chloride, urea, carbon dioxide, glucose and creatinine, respectively. During installation the following parameters were investigated. (1) The maximum deviation between measured values and those declared by the manufacturers ranged between +3·4 and −6·2%. Ten different control sera were used to determine accuracy (300 assays). (2) To investigate the precision, 586 assays were performed. The variation coefficient varied from 0·3 to 3·0%. (3) In 42 assays the carry-over effect was found to be lower than 0·1%. (4) As compared to different routine methods the correlation coefficient was between 0·988 and 0·999 (800 assays).

Astra-8 uses the most recent results of analytical chemistry and data processing. The analyser, with all its built-in programs, is completely satisfactory and reliable for the requirements of modern clinical chemistry.

### 20.2.11: Performance of three kinetic α-amylase methods on the Kone CD clinical analyser

*By G. A. Harff and F. Lamie, Academic Hospital, Free University, Amsterdam, The Netherlands, and Hoechst Holland N.V., Behring Diagnostics, Amsterdam, The Netherlands*

The maltotetraose method of General Diagnostics, the carboxymethylated starch method of Pharmacia Diagnostics and the p-nitrophenyloligosacharide method of Behring Werke were evaluated for use on the Kone CD. The determinations were performed at 30°C, with 5 min. lag time and 3 min. kinetic measurement. Kone D Analysis No. 27·2 was used. The photometric response for Ortho Normal control serum (lot No. 11T222) was 11mA/min. at a volume fraction of the sample of 0·0476 with the 'maltotetraose' method; 14 mA/min. at a fraction of 0·00662 with the 'starch' method; and 13 mA/min. at a fraction of 0·0385 with the 'p-nitrophenyl' method. Patients' sera showed almost the same ratio of mA/min. The volume fractions are according to the manufacturer's instructions. A disadvantage of the 'starch' method is the need to predilute the sample because of the small volume fraction. Linearity and day-to-day precision are presented. The method of comparison was the Phadebas Amylase (tablet) method, in which results are expressed at 37°C. Least squares regression equations were: Phadebas = x(U/l) and kinetic method = y(U/l), $N=60$

'maltotetraose' method: $y=0{\cdot}095\ x-0{\cdot}04$ $r=0{\cdot}973$
'starch' method: $y=0{\cdot}434\ x-0{\cdot}09$ $r=0{\cdot}993$
'p-nitrophenyl' method: $y=0{\cdot}198\ x-1{\cdot}0$ $r=0{\cdot}992$

Interference was found for pyruvate (from 1 mmol/l), α-ketobutaric acid (from 0·3 mmol/l), α-ketoglutaric acid (from 0·5 mmol/l) and $H^+$ (from 20 mmol/l) with the 'maltotetraose' method. Glucose gave an elevated initial absorbance with the 'starch' method. Bilirubin (from 350 μmol/l), $H^+$ (from 10 mmol/l) and EDTA (from 5 mmol/l) interfered with the 'p-nitrophenyl' method. No interference was found for heparin, citrate, magnesia, fluoride, Hb, bromide, phosphate, urea, acetoacetic acid, ethanol, aceton and oxalate.

### 20.2.12: Evaluation of the Beckman Electrolyte 2, an indirect potentiometric instrument for analysis of sodium and potassium in serum and urine

*By G. A. Harff and C. van Leeuwen, Department of Clinical Chemistry, Academic Hospital, Free University, PO Box 7057, 1007 MB Amsterdam, The Netherlands*

The authors evaluated and compared the Beckman Electrolyte 2 to the IL 243 flame photometer. For serum, the Beckman liquid control sera, Decision Levels 1, 2 and 3 were used to assess the within-run and between-day precision.

| Within-run precision | | | |
|---|---|---|---|
| | x | SD | *N* |
| Serum $Na^+$ | 125·8 | 0·5 | 38 |
| | 137·4 | 0·5 | 38 |
| | 149·4 | 1·2 | 38 |
| Serum $K^+$ | 2·73 | 0·01 | 38 |
| | 4·22 | 0·02 | 38 |
| | 5·76 | 0·05 | 38 |
| **Between-day precision** | | | |
| | x | SD | *N* |
| Serum $Na^+$ | 125·7 | 1·1 | 21 |
| | 137·6 | 1·2 | 21 |
| | 151·4 | 1·3 | 21 |
| Serum $K^+$ | 2·68 | 0·02 | 21 |
| | 4·19 | 0·03 | 21 |
| | 5·87 | 0·05 | 21 |

For urine, within-run and between-day precision were assessed with the Hyland Q-Pak Chemistry Urine Control.

| Within-run precision | | | |
|---|---|---|---|
| Urine $Na^+$ | 87·3 | 0·3 | 20 |
| Urine $K^+$ | 30·8 | 0·2 | 20 |
| **Between-day precision** | | | |
| Urine $Na^+$ | 87·3 | 0·9 | 20 |
| Urine $K^+$ | 30·7 | 0·3 | 20 |

Method comparison was performed with the IL 243 flame photometer. Deming's calculation of regression equations were used. Serum $Na^+$: $y(\text{ISE})=1{\cdot}024\ x(\text{flame})+0{\cdot}0$. $S_{yx}=0{\cdot}39$ and serum $K^+$: $y=1{\cdot}024\ x-0{\cdot}093$ $S_{yx}=0{\cdot}12$. For urine mode least squares regression equations were $Na^+$: $y=0{\cdot}9995\ x-0{\cdot}95$, $r=0{\cdot}9997$ and $K^+$: $y=0{\cdot}9838\ x-0{\cdot}15$, $r=0{\cdot}9987$. The $Na^+$ electrode was influenced by the $K^+$ concentration in urine samples. At a concentration for $Na^+$ of 50 mmol/l this effect was $y=Na^+$ and $x=K^+$; $y=0{\cdot}0205\ x+50{\cdot}4$, $S_{yx}=0{\cdot}22$. The higher the $Na^+$ concentration the larger the absolute contribution of $K^+$ to the $Na^+$ result.

**20.2.13: Evaluation of sodium and potassium determinations in serum and urine on KONE OY's Microlyte**

*By G. A. Harff and C. van Leeuwen, Department of Clinical Chemistry, Academic Hospital, Free University, PO Box 7057, 1007 MB Amsterdam, The Netherlands*

Direct potentiometric determinations of the electrolytes sodium, potassium and ionized calcium in whole blood, plasma, serum and urine can be performed on the Microlyte. Dimensions of the compact apparatus are (height, depth, width) 255 × 210 × 315 mm. The authors assessed sodium and potassium in serum and urine. The instrument tested contained PROM version 2·1 NCCLS protocols PSLP-2 and 3 were followed to evaluate the precision. The method of comparison was flame photometry on the IL 143. The regression equations for the serum mode were calculated according to the method of Deming.

Serum Na: $y$(ISE) = 0·866 $x$(flame) + 19·0 $\bar{x}$ = 138·6 $\bar{y}$ = 139·0 $S_{yx}$ = 1·14 $N$ = 85.

Serum K: $y$(ISE) = 0·899 $x$(flame) + 0·42 $\bar{x}$ = 4·31 $\bar{y}$ = 4·30 $S_{yx}$ = 0·059 $N$ = 85.

For the urine mode the samples were manually pre-diluted five times according to the manufacturer's recommendations. Least squares regression equations are presented. Some relevant substances were tested for interference.

**20.2.14: The evaluation of biochemical parameters among the Novi Sad district population, Yugoslavia, by using the Technicon System: SMA II**

*By A. Gavrilović, Lj. Ristić, Z. Radujkov, M. Vučurević, Dj. Krstić and D. Penić, Institute of Pathological Physiology and Laboratory Diagnostic, Medical Faculty, Hajduk Veljkova 1, Novi Sad, Yugoslavia*

During two years' experience of operating the Technicon System SMA II, the authors examined about 25 000 patients from the Novi Sad district in Yugoslavia. The following parameters were measured: iron, creatinine, uric acid, triglycerides, phosphorus, cholesterol, calcium, glucose, total bilirubin, urea, alkaline phosphatase and total protein. At the same time the authors determined the reference values of the above-mentioned parameters and area. In the course of routine operations, the authors have performed permanent, daily quality controls, by using different biochemical control sera.

**20.2.15: Application of innovative technology in a new random access analyser**

*By J. Hamm, 39 Boulevard de la Muette, 95140 Garges-les-Gonesse, France*

New technology for discrete aspiration and delivery of micro volumes of liquids with effective elimination of contamination allows rapid analysis in a random access, while maintaining a high quality of test results. This technology and its application with the new Technicon RA-1000 analyser is described.

Performance data for clinical chemistry methods are also given.

**20.2.16: The Technicon SMAC II C 9000 series**

*By Jean Gaumeton, 39 Boulevard de la Muette, 95140 Garges-les-Gonesse, France*

The Technicon SMAC II is new concept designed to support the analytical and data handling needs of the laboratory. It consists of two major components: the analytical console and data-management package. All analyses are performed automatically from 12 up to 20 chemistries using improved techniques, for example ion-selective electrodes for sodium and potassium, bound enzyme technology for glucose and uric acid, multi-point measurement of enzymatic activity. The samples, identified by a new bar-code identification label, are treated in batches or in stat (for emergency specimens). A new statistical program using Westgard's algorithm checks the accuracy of each result on-line. With the integrated Syslab computer of the SMAC II, the entire laboratory organization including sample or patient reception, analytical process, secretarial tasks and laboratory management is achieved. SMAC II is a major advance in laboratory efficiency.

*Editorial note:* The abstract of poster No. 20.2.17 is not in English and so is not published here.

**20.2.18: Evaluation of a third-generation centrifugal analyser: the Flexigem system**

*By Augusto Corominas Vilardell, Residencia del Insalud and Analysis Laboratory of Badalona, Spain*

After having realized the quality-control of the Gemeni system, integrated in the second generation of centrifugal analysers, the author initiated a preliminary evaluation of the Flexigem system. The following aspects were considered. (1) Precision, at three different levels, of the most common tests made in any laboratory for biochemical analysis (glucose, cholesterol and urea), during a period of three weeks. (2) A study of contamination in tests like creatinine (Jaffé method) calcium/cresolphthalein complexone. (3) Correlation of kinetic results working with three possible analyser temperatures: 25°C, 30°C and 37°C. (4) The use of the calibration curve memory which allows work on parameter log-logit, parameter logit, parameter exponential and parameter polynomial.

The author reports that the results obtained in the evaluation have been satisfactory.

**20.2.19: Dry chemistry: preliminary evaluation of the Seralyzer**

*By Francesco Aguzzi (1), Antonio Montinaro (2), Ferruccio Ceriotti (2), Rossana Colomi (1) and Murone Michelangelo (2), (1) Laboratorio Analisi Ospedale di Broni e Stradella, Italy, (2) Laboratorio di Ricerche Cliniche Ospedale san Raffaele (Istituto di Ricovero e Cura a Carattere Scientifico), 20132 Milano, Italy*

The Seralyzer is a small instrument for dry-chemistry semi-automatic determination of the most relevant biochemical parameters. The paper illustrates the results of a two-centre evaluation trial of the Seralyzer. Glucose, urea, bilirubin, uric acid, cholesterol, creatinine and creatinine-phosphokinase have been studied. After a familiarization period, the precision, accuracy, influence of anticoagulants have been assessed. The results are discussed and the problems of staff training are reported.

### Session 20.3: Systems for Kinetic Measurements

**20.3.1: Determination of free haemoglobin in serum and plasma by an automated kinetic assay**

*By Kurt Bauer, Institute for Clinical Chemistry and Laboratory Medicine, University of Vienna, A 1090 Wien, Lazarettg. 14, Austria*

A kinetic assay for the determination of free haemoglobin in serum as well as in plasma is described. The method is suitable for use in automated systems. Commercially available reagent solutions, containing 4-aminophenazone as chromogen, and haemoglobin cyanide standard solutions were used. Procedures, reference intervals, statistics, and limitations are shown for the determination in serum and heparin plasma. Because of its speed and accuracy, this test is valuable for human and animal applications.

**20.3.2: An automated method for the measurement of serum phosphodiesterase I activity**

*By C. H. Konings, Department of Clinical Chemistry, Academic Hospital, Free University, Amsterdam, The Netherlands*

A kinetic assay for determining phosphodiesterase I (E.C. 3.1.4.1.) in human serum was developed using a Gemsaec centrifugal analyser. 40 $\mu$l of serum and 80 $\mu$l of water were mixed with 320 $\mu$l of reagent consisting of thymidine-5′-monophosphate-p-nitrophenol (T5MN) in a Tris-HCl buffer at pH 9·65. The release of paranitrophenol from the T5′MN by the catalytic action of the enzyme is monitored for 90 s at 405 nm. The assay is carried out at 30°C and is linear for phosphodiesterase I activity to 260 U/l. Within-run precision for a pooled serum sample and a commercial control serum gave CVs of 4·0 and 2·7% (mean values of 35 and 69 U/l respectively). A comparison study between the presented method and a manual assay using the same substrate is discussed. Results of scientific studies on patients' sera using this rapid and sensitive automated technique are given.

### 20.3.3: Automated assay of serum acid phosphatase and its use in the diagnosis of prostatic carcinoma

*By H. Caillens, T. D. Nguyen, R. Chiche, O. G. Ekindjian, L. Boccon-Gibod and J. Yonger, Laboratory of Clinical Chemistry and Urology Department, Cochin Hospital, Paris, France*

A kinetic method of serum acid phosphatase determination has been adapted to the Cobas Bio centrifugal analyser, using α-naphtylphosphate as a substrate and concurrent diazotation of the released α-naphtol with fast red TR. The prostatic acid phosphatase activity is inhibited by L(+) tartrate. The time necessary for the test was 6 min. 30 s. Under the conditions described the response was linear up to 340 UI/l, repeatability and reproducibility were good. The results were compared with those obtained by a manual method using p-nitrophenylphosphate as substrate. There was a close correlation between the two methods for both total acid phosphatase ($r = 0{\cdot}99$) and prostatic acid phosphatase ($r = 0{\cdot}97$). The diagnostic value of the proposed method for the detection of prostatic carcinoma has been tested on selected patients in the urology department at Cochin Hospital. A preliminary study on 86 subjects showed no false positive results. The relation between acid phosphatase activities and the clinical stages of the disease are discussed for 21 cases of confirmed prostatic carcinoma.

### 20.3.4: Amniotic fluid cholinesterase measurement with a centrifugal analyser

*By F. Moreau, C. Guermeur, F. Papa, O. G. Ekindjian, R. Henrion and J. Yonger, Cochin Hospital, Paris, France*

Amniotic fluid total cholinesterase (TChE) and acetylcholinesterase (AChE) activities are usually measured for the exclusion of neural-tube defects. Ellman based determinations of TChE on the hydrolysis of acetylthiocholine and detection of the free thiol released by DTNB (5-5′ dithiobis [2-nitrobenzoic acid]); for a direct assay of AChE, and Dale proposed ethopropazine hydrochloride ('Lysivane'), as an inhibitor of non-specific cholinesterase. A method has been adapted for a Cobas Bio analyser. The sample volume for each determination is 20 μl. TChE is measured for 6 min. 30 s at 30°C in the presence of the substrate after a 1 min. pre-incubation with DTNB in a phosphate buffer at pH 8·0. For AChE, 1 min. pre-incubation time with 14μm inhibitor has been considered as optimal, following preliminary studies with 1·4 μm to 28 μm Lysivane for 30 s to 300 s. The reactions are linear up to 200 UI/l. Within-run (CV < 3%) and between-run (CV < 5%) precisions are excellent. Non-NTD amniotic fluids were used to establish usual values. The technique proposed is easy, simple, and rapid enough to provide results during the course of an amniocentesis clinic.

### 20.3.5: The bichromatic determination of triglycerides on the Multistat III microcentrifugal analyser

*By Claudio Musitelli, Antonio Montinaro, Michelangelo Murone and Pierangelo Bonini, Laboratorio di Ricerche Cliniche, Ospedale San Raffaele, 20132 Milano, Italy*

The Trinder reaction has been widely applied in clinical chemistry in the last few years. This procedure was introduced for the automatic determination of triglycerides on the Multistat III microcentrifugal analyser. The presence of surfactants in the reaction mixture, and the peculiar design of the Multistat rotors, can result in a premature mixing of sample and reagents, thus preventing the use of the fixed-time procedure. The Multistat III's software requires at least two readings, at two different times or at two different wavelengths (bichromatic procedure). The authors adopted the end-point bichromatic procedure and the results compare well with those obtained by the manual determination.

### 20.3.6: The use of a bacteric cholesterol-esterase for the automatic determination of cholesterol

*By Pierangelo Bonini, Claudio Musitelli, Ferruccio Ceriotti and Michelangelo Murone, Laboratorio di Ricerche Cliniche, Ospedale San Raffaele, 20132 Milano, Italy*

The enzymatic determination of cholesterol using commercial kits requires from 10 to 12 min. incubation at 37°C, for full development of the colour. The incubation time can be reduced by increasing the amount of the pancreatic cholesterol-esterase in the reaction mixture (which increases the cost of the determination), or by replacing it with a more active one. By introducing a bacteric cholesterol-esterase, the 37°C incubation time was reduced to 4 min. This method has been successfully applied to the Multistat III microcentrifugal analyser with a bichromatic procedure. The results correlate well with those obtained by the manual procedure.

### Session 21.1: Microbiology

### 21.1.1: Laboratory evaluation of the Kirby-Bauer Disc diffusion test and the Autobac-I system

*By E. Boquet, C. Romeu, D. Xairó and J. L. Artigalás, Centro Hospitalario de Manresa, C/ Bases 6y8, Manresa, Spain*

The results obtained with the standardized disc diffusion test were compared to those obtained by an automated susceptibility testing system, the Autobac-I (Pfizzer). The comparative investigation involved over 517 micro-organisms isolated from clinical specimens. The antimicrobial agents tested were: nalidixic acid, amikacin, ampicillin, carbenicillin, cephalosporin, chloramphenicol, cloxacillin, colistin, sulfamethoxazole + trimethoprim, erytromycin, gentamicin, nitrofurantoin, penicillin, tetracyline, and tobramycin. The similarities and differences of the methods were investigated. Discrepancies were classified as (*a*) very major — susceptible by Autobac I, resistant by disc diffusion; (*b*) major—resistant by Autobac I, susceptible by disc diffusion; (*c*) minor—intermediate in one of the two methods.

The overall average for the 15 antimicrobial agents tested was 91·96%—nalidixic acid was 95·14%, amikacin 82·74%, ampicillin 82·21%, carbenicillin 95·38%, cephalosporin 91·65%, chloramphenicol 100%, cloxacillin 92·86%, colistin 97·26%, sulfamethoxazole + trimethoprim 92·80%, erytromycin 97·27%, gentamicin 97·01%, nitrofurantoin 88·82%, penicillin 77·17%, tetracycline 96·86% and tobramycin 92·46%.

The values of medium discrepancies were: very major 1·27%, major 2·16% and minor 4·61%.

### 21.1.2: An evaluation of the Replireader for the identification and sensitivity determination of Gram negative bacilli

*By G. Mascart, G. Gazon, and Ph. Mascart, Department of Clinical Pathology, Edith Cavell Clinic, rue Edith Cavell 32, 1180 Brussels, Belgium*

The authors present an evaluation of the Replireader, a new semi-automatic system for identification and antibiotic susceptibility determination of Gram negative bacilli. The basic principle of this system is replication on agar plates. 17 biochemical plates and 22 antibiotic plates were used, and 641 Gram negative bacilli, most of which were Enterobacticereae were tested. 94·5% of identifications were correct. 2·34% of the strains were not identified and 3·12% were misidentified. In this last category half of the strains were misidentified only as far as the species, and not the genus, were concerned. The antibiotic susceptibility results were in close agreement with those obtained with the Kirby-Bauer method. Indeed, there was a 97·1% agreement between the two methods. Minor discrepancies amounted to 1·1% and major discrepancies 1·7%. The Replireader appears to be an excellent alternative to conventional methods. It is very easy to use. Quality of work can be higher than with conventional methods as a result of the systematic quality control of the plates and more complete biochemical data is used. One single inoculum is used for identification and susceptibility determination. It is possible to save time, media and reagents.

**21.1.3: Rapid automated susceptibility testing with the MS-2 system**

*By G. Szilagyi and V. Aning, Hospital of the Albert Einstein College of Medicine, Bronx, New York, USA*

Early reporting of antimicrobial susceptibility test result of bacterial isolates from clinical specimens is an important factor in the better care of hospitalized patients. Several semi-automated and automated instruments have been developed for rapid susceptibility testing. The MS-2 microbiology system (Abbott Laboratories) is a computerized automated system which performs this analysis by photometrically monitoring turbidimetric changes that result from bacterial growth. The MS-2 system was evaluated for the accuracy of the antimicrobial susceptibility test results. A total of 482 clinical isolates (396 gram negative and 86 gram positive) were tested and the results compared with those obtained from the conventional disc diffusion method. Discrepancies were categorized as very major, major and minor. Agreement was determined by organism and by antimicrobial agent. Full accord (all discrepancies considered) of 96·6% and essential accord (minor discrepancies excluded) of 98·1% was found with gram negative organisms. With gram positive organisms 84·0% full accord and 92·9% essential accord was observed. Mean test time for all isolates was 4·2 h. Susceptibility testing with the MS-2 system was found to be an accurate and rapid method, readily adaptable to the work flow in the authors' clinical microbiology laboratory.

**21.1.4: Comparative study of antibiotic sensitivities of bacteria with manual and automated methods**

*By László Salgó, Ervin Szöllösi and Erzsébet Nagy, Department of Paediatrics, Department of Clinical Microbiology, Central Laboratory, University Medical School, Szeged, Hungary*

Only during the past few years has automation made a significant impact in the clinical bacteriology laboratory. The ABAC (Sclavo, Siena, Italy) automatic system was tested. A 4 h broth-culture was prepared from the bacteria isolated from 400 clinical patients; this served as the standard starting material for the various methods. Depending on the bacteria isolated, the sensitivity was examined for four different antibiotic or chemotherapeutic groups. The disc-diffusion test method generally employed in Hungary was compared with the results given by the ABAC system, and with the micro-organism drug-sensitivities determined under quantitative conditions; the latter were also obtained by micro and macro procedures. The results were evaluated, and the applications of the methods and the advantages of the automatic technique were assessed.

**21.1.5: Antimocrobial susceptibility testing of *Streptococcus pneumoniae* by two methods**

*By J. L. Pérez, J. Liñares, M. C. Marne and S. Romero, Servicio de Microbiología, C. S. 'Príncipes de España', Hospitalet de Llobregat, Barcelona, Spain*

Thirty clinical isolates of *S. pneumoniae* were tested for susceptibility to penicillin, ampicillin, tetracycline, chloramphenicol, erythromycin and clindamycin by the standard agar dilution method. Results were compared to those obtained using a commercial microdilution product, Sensititre, in which Todd-Hewitt broth was supplemented with 5% defibrinated whole horse blood. Among the 30 strains, two were resistant (MICs 2 mcg/ml), two were relatively resistant (0·12–0·5 mcg/ml) and 25 were susceptible to penicillin by both methods. Only one strain showed a three dilutional step difference by microdilution broth and agar dilution, resulting in categorization as susceptible by the former method but relatively resistant by the latter.

The Sensititre and agar dilution minimum inhibitory concentrations (MICs) were equivalent within ±1 dilution in 96% of the comparable test results for penicillin, ampicillin and chloramphenicol. The correlation was excellent for erythromycin, clindamycin and tetracycline.

The micro-broth dilution technique is a reliable, simple and convenient semi-automated method of determining MICs of *S. pneumoniae*, with the advantage of an extended shelf-life when stored at room temperature.

**21.1.6: Microdilution aminoglycoside susceptibility testing of *Pseudomonas aeruginosa* with divalent cation supplemented inoculum**

*By M. C. Marne, J. Liñares, A. Gasós, N. Parrero and J. L. Pérez, Servicio de Microbiología, C. S. 'Príncipes de España', Hospitalet de Llobregat, Barcelona, Spain*

The concentration of magnesium and calcium in media has been cited as a factor affecting susceptibility results of aminoglycoside testing of *P. aeruginosa*. The purpose of this study was to compare the minimum inhibitory concentrations (MICs) obtained for three aminoglycosides (gentamicin, tobramycin and amikacin) by the sensititre technique using unsupplemented and supplemented inoculum, with the agar dilution MICs.

Thirty one clinical strains of *P. aeruginosa*, previously checked as aminoglycoside susceptible by the disc diffusion method, were used. Tests were run in parallel with Mueller-Hinton broth unsupplemented and supplemented with 25 mg of magnesium and 50 mg of calcium/l. Agar dilution MICs were determined by conventional methods. The susceptibility in supplemented broth had 100% agreement (± one doubling dilution) with agar dilutions for the three aminoglycosides. With the unsupplemented broth *P. aeruginosa* was found to be more susceptible than with the agar dilution method. Differences ranged from two to four doubling dilutions for gentamicin, two to four for tobramycin and one to three for amikacin.

Divalent cation content of the testing media must be controlled for valid comparison between antipseudomonal antibiotics. The use of the sensititre microdilution procedure for MICs determinations with supplemented broth compares well with standard agar dilution methods, but is simpler to perform.

**21.1.7: Evaluation of automated procedures for monitoring antibiotic treatment of acute bacterial infections**

*By A. Visconti and U. Marini, Laboratory of Clinical Microbiology and Department of Internal Medicine VI, Ospedale S. Carlo Borromeo, Milan, Italy*

The outcome of treatment in acute bacterial infections is very dependent on the rational choice of proper antimicrobial agents. For that purpose previous susceptibility testing of clinical isolates should be mandatory whenever possible. The most useful and important information from such testing is expected when several parameters, such as minimal inhibitory (MIC) and bactericidal (MBC) concentrations, MBC:MIC ratio (or tolerance), killing rate, growth curves inhibition, drug level in body fluids, bacterial activity of the host serum and opsonic requirements, are simultaneously evaluated at the right moments during therapy.

A number of patients with acute bacterial infections (pneumonia, meningitis, sepsis, pyelonephritis etc.) were studied by testing all these parameters using automated procedures, such as turbidity (MS-2 Research System) and bioluminescence (Luminometer) methods. The effects of inoculum, media, time of incubation, and other experimental variables were also evaluated.

The results are presented and discussed by the authors.

### Session 21.2: Clinical Chemistry Immunoassay

### 21.2.1: Comparision of automated and manual techniques for the determination of creatine kinase-MB

*By J. P. Chapelle, Department of Clinical Chemistry, University of Liège, 1 rue des Bonnes-Villes, B 4020 Liege, Belgium*

The immuno-inhibition method for the determination of creatine kinase-MB (CK-MB) activities has been automated by the author and the results have been compared with those of two manual techniques: ion-exchange chromatography and polyacrylamide gel electrophoresis (PAGE). The precision of the automated technique was evaluated through the measuring range of CK-MB, providing values of 0·8 to 4·3% CV. The linearity limit was 250 IU/L.

The automated immunological method correlated well with the chromatographic technique ($r = 0{\cdot}98$). For some serum specimens, however, the two techniques led to conflicting results, the immunological method indicating high CK-MB values (>15% of total CK) whereas activities determined after chromatographic separation were within the normal range. PAGE indicated in those cases the absence of CK-MB and the presence of abnormally migrating CK-MM sub-bands. These forms were not inhibited by the anti-CK-M antibodies and were therefore responsible for an apparent CK-MB activity when the immunological technique was used. The abnormal CK-MM sub-bands were not associated with myocardial damage.

The automated immuno-inhibition technique offers advantages of time saving, increased precision and accuracy, and is easily adaptable to the laboratory routine. However, when elevated CK-MB activities (15–30% of total CK) are found, the presence of this isoenzyme must be confirmed by electrophoresis.

### 21.2.2: Automated RIA determination ($^{125}$J) of serum thyroxine concentration and $T_3$-binding capacity

*By S. Knapp and N. Panitz, Medizinisch Diagnostisches Institut Dr Kapp, 6500 Mainz, FR Germany and Deutsche Klinik f. Diagnostik, FB Nuklearmedizin, 6500 Wiesbaden, FR Germany*

To reduce the difficulties with incubation and separation of mechanized RIA tests a new kit was developed. After incubation in a continuous-flow system the bound fraction was separated by magnetification of antibody-carrying particles (Technicon STAR). The bound fraction of the $^{125}$J-labelled antigen was then measured.

Precision, accuracy and correlation were the same as in a manual test-kit (PEG separation). The inter-assay coefficients of variation ranged from 3·1% to 5·3% for thyroxine and from 4·6% to 11·6% for $T_3$-binding capacity. The normal range was identical to that of the manual test-kit.

The results obtained for serum thyroxine concentration and $T_3$-binding capacity from more than 200 patients were compared with all others *in vitro* (by the TRH stimulation test); in addition, clinical *in vivo* investigations were made. Significant differences were found among the groups with euthyroid goiter without and under oestrogen medication, pregnancy, treatment with thyroxine, normals, hyper- and hypothyroid diseases. To evalute the diagnostic validity of the results the authors compared the percentage deviation from the normal range within each group.

Although the determination of thyroxine, $T_3$-binding capacity and $FT_4$-index make it easy to recognize diseases, further *in vitro* and *in vivo* parameters should be investigated in some cases.

### 21.2.3: An automated enzyme immunoassay for serum ferritin

*By T. Carter, R. Bunce and T. P. Whitehead, Wolfson Research Laboratories, QEMC, Birmingham, UK*

'Sandwich' enzyme immunoassay (EIA) is becoming an increasingly popular alternative to radioimmunoassay. When performed with polystyrene tubes or micro-titration plates as the solid phase, precision is often unacceptably poor and, moreover, these are unsuitable for automation. An EIA system using antibody-coated polystyrene balls and a specially-designed incubation module for use together with components of the Kone CD Parallel Analyser has been developed. The system is versatile and allows the simultaneous analysis of 96 specimens in approximately 3 h. In an assay for serum ferritin, the detection limit was about 1 ng/ml with a working range up to 300 ng/ml and a precision of 6·5% at 100 ng/ml.

### 21.2.4: Automated labelled antibody immunoassays

*By Heather A. Kemp, Rhys John and J. Stuart Woodhead, Department of Medical Biochemistry, Welsh National School of Medicine, Cardiff, UK*

A range of labelled antibody assays for use on a fully automated microprocessor-controlled immunoassay system (Kemtek 3000) has been developed. These automated assays are capable of rapidly processing large numbers of samples. Examples of assays where this technology has proved particularly valuable are those of $\alpha_1$-fetoprotein (AFP) and thyrotropin (TSH), which are both used extensively in population screening. Both are measured by the two-site method in which serum samples are reacted either simultaneously or sequentially with excess labelled antibody and solid phase antibody. Radioactivity bound to the solid phase is a function of the concentration of antigen present. Using a cellulose-linked antibody and $^{125}$I-labelled antibodies, a sensitive AFP assay (detection limit 6 KU/l) can be obtained with a 1 h reaction, followed by separation of the solid phase by means of filtration. Up to 100 patient samples can be processed in a total of 3 h. A screening test for congenital hypothyroidism has been developed which uses 6 mm discs of dried blood on filter paper from neonates. Samples are reacted overnight with $^{125}$I-labelled antibody and then for a further 2 h with cellulose-linked antibody, and finally subjected to automated filtration. The assay has a detection limit of 3·75 $\mu$/l and a CV of only 6% at a TSH concentration of 30 $\mu$/l. Of 14 500 samples screened so far, only six repeats have been necessary; of these two were confirmed cases of hypothyroidism. The flexibility of the Kemtek 3000 and the advantages of labelled antibody technology make the procedures suitable for high-throughput rapid-turnround immunoassays.

### Session 21.3: Immunology

### 21.3.1: Circulating immunocomplexes. Contribution to a new automated technique for their quantification

*By F. L. Elorza, F. Malagon, M. E. Dorado and A. Moreno, Dpto. Bioquimica Clinica, Hospital Universitario de Sevilla, Spain*

Nephelometry is a turbidimetric technique, which is based on the dispersion of a beam of light when it falls on a precipitate dispersed in a liquid. Quantification of circulating immunocomplexes is very useful for the diagnosis and follow-up of a great number of pathologies (hepatic disease, collagen disease etc.). Adopting a low-cost automated technique for this type of quantification would be of great utility to patient care in large hospitals. The authors present their experience with 1300 patients, classified according to their pathologies, and correlate the results of the technique with Bach's manual technique.

### 21.3.2: Determination of alpha-fetoprotein (AFP) in the serum of patients with psoriasis vulgaris by the particle counting immunoassay (PACIA)

*By W. Krotz, A. Leek, Angelika Welter and V. Hochstein, Heinrich-Pette-Institut für Experimentelle Virologie und Immunologie an der Universität Hamburg, Martinistrasse 52, D 2000 Hamburg 20; and Technicon Chemicals Company, Animalerie Centrale 56.20, Avenue Hipocrate 56, B 1200 Brussels, Belgium*

PUVA treatment with psoralen (P) and long-wave ultra-violet light (UVA) of psoriasis vulgaris is associated with an incalculable long-term risk. For example, a certain structural similarity exists between photosensitiziers of this type and the aflatoxins. This gave rise to an investigation of the AFP concentration in the serum of psoriasis patients, employing the PACIA system developed by A. Leek and using modified DAKO antisera. The principle behind this new technique is the agglutination of coated latex particles with the corresponding immunochemical reactant. The latex particles were coated with antibodies of AFP. PACIA is a continuous-flow analysis with an automatic particle count before and after the immunochemical reaction. Using this assay, AFP concentration in the serum of more than 100 psoriasis patients was examined. Patients who had received true PUVA long-term treatment (at least 10 years) were not included. The PACIA-AFP values of health subjects were up to 20 ng/ml; on average these were approximately 10 ng/ml. In contrast, one-third of the psoriasis patients examined had values of 20 to 350 ng/ml. The study will be continued with long-term controls of psoriasis patients treated with PUVA.

### Session 21.4: Clinical Chemistry—Chromatography

### 21.4.1: Liquid chromatographic assay of some anthraquinone glycosides

*By O. O. Komolafe, Department of Pharmaceutical Chemistry, University of Ife, Ile-Ife, Nigeria*

The current methods of assay of some anthracene purgative drugs, which determine both the free and combined anthracene derivatives, lack sensitivity and specificity because the main pharmacological activity of the anthracene derivatives is shown by the glycoside form. This work reports paired-ion chromatographic analysis of the major glycosides of senna pod and some pharmaceutical preparations of senna using a Corasil $C_{18}$ column with methanol (30% in water) containing 0·005 M tetrabutylmmonium phosphate (at pH 7·5) as solvent. Liquid chromatographic analysis of the same crude drug and pharmaceutical preparation therefrom have been carried out using a weak anion-exchange resin with ammonium nitrate solution (0·1 M, pH 7·5) as the mobile phase. These systems allowed for the determination of sennosides A and B in nanogram quantities, free from other adjuncts and excipients present and without derivatization and its inherent problems. The chromatographic methods used in this work cut the assay time for the drugs to about half that required by current assay procedures, and they are recommended for use in automatic systems.

### 21.4.2: Significance of CK values in an adult and paediatric population seen in a university medical centre

*By Horacio A. Perez, Ronald Busch, Gary E. Blank, and Ajit Sanghvi, Clinical Chemistry Laboratory, University Health Center of Pittsburgh, 203 De Soto Street, Pittsburgh, PA 15261, USA*

The central laboratory of the authors' health centre has been, for several years now, measuring levels of total CK and CK isoenzymes in serum with the ABA-100 bichromatic analyser (Abbott Laboratories) at 30°C. Isoenzyme separation is obtained using ion-exchange chromatography columns based on Mercer's method. A retrospective study was conducted comprising 342 patients consecutively admitted to the hospitals during a six-month period, in whom abnormally elevated serum levels of total CK activity were present on admission and/or on subsequent days. A total of 107 patients (31%) comprised the paediatric group admitted to the Children's Hospital. Elevated CK-MM isoenzyme levels were present in accidents involving trauma to skeletal muscle or brain. Elevated CK-MB levels in this group were seen in patients with Duchenne's muscular dystrophy, Reye's syndrome, and patients who had undergone complete liver transplantation. One patient with biliary atresia showed increased CK-BB activity in the serum. The adult patient group (69%) consisted largely of patients admitted to the Coronary Care Unit with suspected and/or diagnosed acute myocardial and/or subendocardial infarction, or infarct extension. Other cases in which CK-MB activity was also demonstrated include patients with dermatomyositis, and patients who had undergone complete liver transplantation. Finally, patients in whom elevated total CK activity was present in the serum without concommitant elevation of CK isoenzymes were found to have pulmonary embolism, true angina pectoris, severe pneumonia, pericarditis, cerebrovascular accidents, general surgery and cardiac catheterization. Also, cases of cardiac defibrillation and intramuscular injection were identified.

### Session 21·5: Mathematics—Clinical Chemistry

### 21.5.1: An automated system of quality control

*By Wieslaw Piechota, Norbert Symonowicz and Wojciech Minta, Centre of Postgraduate Medical Education, 00-909 Warsaw 60, Szaserow 128, Poland*

No single method of quality control is capable of detecting all possible errors which may affect laboratory diagnostic information. Therefore several methods should be used routinely. Such a task, however, poses a great burden on a laboratory. In order to ease this task, several basic methods of quality control were combined into one coherent system using a minicomputer. These methods are based on: (1) results of measurements of control specimens; (2) very unlikely and absurd values; (3) averages of patients' normal results (for a given number or batch) and for daily values; (4) comparison of a result with a patient's previous result; and (5) physiological and pathological correlation of results of the same patient. The algorithms used in the system, with the criteria of correctness of results, are presented. The results to be checked are listed with marks indicating which of the criteria is (are) not met. The relation between the methods employed are discussed (statistical and arbitrary nature of some accepted criteria, for example precision and comparison of the same patient's results etc.) as well as the advantages for technicians and chemical pathologists.

### 21.5.2: Indirect establishing of reference intervals—is its value always questionable?

*By Wieslaw Piechota, Norbert Symonowicz and Zofia Pastusiak, Centre of Postgraduate Medical Education, 00-909 Warsaw 60, Szaserow 128, Poland*

The introduction of an operational term 'reference intervals' instead of an idealistic and abstract 'normal ranges' has revived the idea of seeking indirect methods to compute reference intervals. However, despite a few attempts, the simplest and the oldest method utilizing probability paper has not yet been evaluated completely. This method was evaluated taking into account the following conditions: (1) application of the method to populations which were supposed to be 'healthier' than hospital patients, namely subjects who underwent preventive check-ups ($N=1606$) and ambulatory patients ($N=1768$); (2) application of the method to Technicon SMA 12/60 profiles to avoid bias due to selection of a test by a physician; (3) taking into consideration demographic factors by establishing sex and age dependent ranges; (4) analysing (prior to establishing reference ranges) frequency distributions of 11 constituents in healthy subjects by means of the chi-square test and coefficients of skewness and kurtosis and using transformations if necessary. According to Cherian's criterion, which is based on precision of analytic methods, the reference ranges obtained with the indirect method were no different from the intervals established in the conventional way for 875 healthy individuals.

### 21.5.3: Quality control of densitometer measurements in protein assay

*By A. Lopez, F. Lema, R. Perez, S. Bandin and R. Alvareg, Laboratorio de Bioquímica, R.S. 'Almirante Vierna', Vigo, Spain*

For the measurement of serum protein profiles, commercial control material was used to investigate the within-day and the between-day reproducibility of the complete procedure and of the densitometry component. Linearity and material stability were also studied.

(1) Densitometry accounted for approximately half of the total CV.
(2) The between-day CVs for the whole procedure and for the densitometry component are both increased proportionately compared with corresponding within-day CVs.
(3) Linearity for the different protein fractions is not the same.
(4) When the reconstituted control material is kept at room temperature and at 4°C, the CVs remained within expected limits over the time of the study.

### 21.5.4: Longitudinal study of 20 biochemical parameters (SMAC) in children

*By P. Gomez, C. Coca, C. Vargas and A. Martinez, Servicio de Bioquimica: Hospital Materno-Infantil, C.S.S.S., 1° de Octubre, Ctra. de Andalucia Km 5.3 Madrid, Spain*

Serum samples were taken from 1800 children attending the out-patient clinics at the Primero de Octubre Maternal-Child Hospital from July 1980 to October 1981. The children's ages ranged from a few days old to 14 years and they were grouped by year. The following were determined simultaneously for each serum: glucose creatinine, uric acid, inorganic phosphorus, calcium, sodium, potassium chloride, iron, cholesterol, triglycerides, total protein, albumin, total bilirubin, creatinphosphokinase, lactic dehydrogenase, alkaline phosphatase, total carbon dioxide, alanine aminotransferase, and aspartic aminotransferase in a continuous flow autoanalyser (SMAC).

The statistical method includes codification of data and processing on an IBM 370, carrying out a transverse study of each of the biochemical parameters, analysis of respective distributions, and elimination of outlying observations by the Grubbs method. Likewise, a percentile study has been performed on each of the distributions, followed by longitudinal adjustment of the same to polynomic functions. Results and conclusions are presented.

### 21.5.5: Measurement of creatinine by continuous flow. Contribution of statistics to obtaining accuracy

*By O. Houot, L. Lepage and R. Gueguen, Centre de Médecine Préventive, 2 Avenue du Doyen Jacques Parisot, 54500 Vandoeuvre-les-Nancy, France*

The measurement of creatinine in plasma or serum by Jaffé's method is known to be affected by numerous interferences. Many modifications of the method have been proposed to partially eliminate the reaction of other endogenous compounds with the picric acid. The authors discuss a statistical method which enables the calculation of known interferences due to protein, glucose and urea. The reference method used for this calculation is based on the specific adsorption of creatinine by Lloyd's reagent (Haeckel). The formula is based on the correlation coefficients measured between the different constituents, and the results obtained by calculation are shown to be comparable with those of the reference method. The formula is applied to results determined for creatininemia on the SMA II (Technicon) on supposedly healthy subjects. The authors evaluate the relationship between the calculated creatinine and physiological parameters (height and weight for example).

### Session 21.6: Mathematics—Haematology

### 21.6.1: Erythropoietic model in evaluating hypokinetic effects

*By Y. G. Zorbas, Yu. M. Portnoy and M. D. Dilek, Life Science Division, European Institute of Environmental Cybernetics, Athens 514/1, Greece*

Modelling the system of erythropoiesis is a basic component in presenting or describing processes connected with an organism's reliability (adaptive capacity) to hypokinesia (HK) (limited motor activity) or weightlessness. The aim of this report is to describe a mathematical model of erythropoiesis and its regulation, which takes the age structure of the red blood cell population into account. A flowchart of the model containing the principal elements and factors was prepared. Biological and medical concepts were applied in order to construct the flowchart. It was concluded that this mathematical model can be considered in evaluating the influence of prolonged hypokinetic conditions, both in examining the state of the erythropoietic system proper and describing a certain set of models that simulate the integral effect of hypokinesia on the organism.

### Session 22.1: Haematology Evaluation

### 22.1.1: Comparative study between the hematimetric values obtained with automatic methods (Haemalog 8/90 and Coulter S systems) and manual methods

*By A. Viloria, M. Fernández de Castro, C. Larrocha, M. C. Jiménez Herráez, A. Fernández Zamorano, A. Sicilia and J. L. Fernández-Chacón, Departmento de Laboratoria, C.S.S.S. 'La Paz', Madrid, Spain*

Values obtained for leukocytes, red cells, haemoglobin and packed cell volume with a Hemalog 8/90 system are compared with the results obtained with a Coulter S system. 204 samples were processed for the leukocyte study, the correlation index was 0·97 and the linear regression line was $y=0{\cdot}35+0{\cdot}98\,x$ (where $y$ = values obtained with the Coulter S system and $x$ = values obtained with the Hemalog 8/90 system). 203 samples were used for the red cell study, the correlation index was 0·90 and the linear regression line was $y=0{\cdot}03+0{\cdot}97\,x$. 207 samples were used for the haemoglobin study, the correlation index was 0·99 and the linear regression line was $y=0{\cdot}16+1{\cdot}01\,x$. 200 samples were used for the packed cell volume study, the correlation index was 0·92 and the linear regression line was $y=1{\cdot}03+0{\cdot}96\,x$. Also, the values for haemoglobin and packed cell volume obtained with a Hemalog 8/90 system were compared with the values obtained by manual methods (cianmetahaemoglobin and microhematocrit). 95 samples were used in the haemoglobin study, the correlation index was 0·99 and the linear regression line was $y=0{\cdot}12+0{\cdot}97\,x$. 93 samples were used in the packed cell volume study, the correlation index was 0·99 and the linear regression line was $y=1{\cdot}46+1{\cdot}01\,x$.

There is a good correlation between all the parameters studied, especially in the case of haemoglobin (Hemalog 8/90 versus Coulter S and Hemalog 8/90 versus manual methods), in the case of leukocyte (Hemalog 8/90 versus Coulter S) and that of packed cell volume (Hemalog 8/90 versus manual methods).

### 22.1.2: Comparison between four automated systems for leukocyte differential count

*By J. M. Jou, J. Ll. Vives-Corrons, J. Ll. Aguilar, M. J. Insa, A. Ester, J. Del Olmo and E. Colomer, Laboratori Central*

*d'Hematologia i Escola Professional d'Hematologia 'Farreras Valenti', Hospital Clinic i Provincial, Universitat de Barcelona, Barcelona, Spain*

Four automated systems for leukocyte differential count were evaluated: (*a*) Hematrak-360, (*b*) Diff-3, (*c*) ADC-500 and (*d*) Hemalog D/90. In systems *a*, *b* and *c* an automated leukocyte differential count was based on morphological interpretation from Wright stained smears, and in system *d* it was based on flow cytochemistry and size analysis of EDTA-treated whole blood. Blood samples were obtained from 890 ambulatory adults and 1080 hospitalized non-haematologic patients. The manual counts were interpreted by four technologists over a period of 20 months. For each instrument the parameters studied were: (1) sample preparation for analysis; (2) reproducibility (*c*); (3) correlation coefficient (*r*) between the automated differential counters and the manual method; (4) comparison of the variations in percentage for all cell classes detected by the manual method versus the automated differential counters. In instruments *a* and *d* red blood cell morphology and semi-quantitative platelet appreciation were also studied.

The results obtained showed good *c* and *r* for neutrophils and lymphocytes in all systems analysed but deficient for 'bands' (given by instruments *a*, *b* and *c*) and monocytes. Sample preparation was cumbersome in system *b* and differences in all cell class percentage between count and automated systems varied from 10·5% to 25%.

### 22.1.3: Evaluation of the Technicon H600: preliminary data on utilization and significance of the red cell distribution width (RDW)

*By C. Izzo, L. Miano and A. M. Ferrari, Centro Diagnostico Italiano, Via Saint Bon 20, 20147 Milan, Italy*

Around 80 000 whole blood counts and leucocyte differential counts are performed at the Centro Diagnostico Italiano each year. A Technicon H6000 was acquired in 1981. The instrument determines the seven conventional haematological parameters (WBC, RBC, HGB, HCT, MCV, MCH, MCHC), leucocyte differential count, platelet count and indices (mean platelet volume and plateletcrit), size histograms and distributional width for red cells and platelets simultaneously on each blood sample. Analyser imprecision and correlation between H6000 and Coulter S results were evaluated. Clinical utilization and significance of the red cell distribution width (RDW) in some haematological disorders was also studied.

Imprecision was determined for low, medium and high values of all parameters, testing four replicates of 60 blood specimens each day for 20 days. Results obtained in all three groups of values showed a CV lower than 2·8% for conventional haematological parameters, 3·6% for leucocyte differential count, 6·6% for platelet count and indices. Data on correlation between the H6000 and Coulter S, analysed by the Deming's method, showed a correlation coefficient ranging from 0·907 to 0·960. Clinical significance of RDW was evaluated on 65 patients with B-Thalassemia (TH), 62 with iron deficiency (ID), 61 with miscellaneous anaemia (MA) in comparison with 1500 normal controls (NC). Results were respectively: $23{\cdot}2 \pm 1{\cdot}4$ for TH, $19{\cdot}3 \pm 1{\cdot}6$ for ID, $15{\cdot}9 \pm 2{\cdot}1$ for MA, $16{\cdot}5 \pm 1{\cdot}0$ for NC. To investigate the significance of these findings a study on correlation between RDW and other parameters (MCV, osmotic fragility, serum iron and % Hb A2) was carried out. A good negative correlation was observed only with MCV and osmotic fragility ($r = -0{\cdot}907$ and $-0{\cdot}894$ respectively).

The Technicon H6000 is shown to be a reliable instrument, which, despite technical differences, shows good correlation to Coulter S. The RDW index on the H6000 seems to be related to red cell distribution width as well as to red cell mean volume; this feature makes it particularly suitable for some clinical purposes.

### 22.1.4: Evaluation of Technicon H6000 haematology system to handle all routine haematology tests in a rapid, accurate and cost-effective manner

*By P. W. Stiffler and C. Joson, Damon Clinical Laboratories, Inc., 4720 W. Montrose Ave., Chicago, Illinois 60641, USA*

The Technicon H6000 system is a one work-station, fully automated haematology analyser that provides in a single report the RBC, WBC, Hgb, HCT, MCV, MCH, MCHC, platelet count, platelet size histogram, red cell size histogram and a 10 000 cell WBC differential (both absolute numbers and percentages of leukocyte population) plus a permanent monolayer slide all from a 0·55 ml whole blood sample. Samples are processed at a rate of up to 90/h with lag time of 10 min. by flow-through cytometry with electro-optical counting and sizing technology plus cytochemical staining.

For the 400 samples analysed, the overall correlation for the H6000 CBC and platelet counts with the Coulter $S^+$ PLUS was 0·95. The correlation for the H6000 10 000 cell differential with the standard manual 100 cell differential was 0·85 for polymorphonuclear leukocytes and lymphocytes for the 160 normal samples analysed. This was mainly due to the imprecision of the manual counts. Precision studies on the H6000 on duplicate samples was far superior to the manual readings. The H6000 system is reliable and accurate for screening out the normal human blood samples in a patient population (80%) so that only the specimens having blood cell abnormalities (20%) need to be screened by haematologists and/or pathologists.

Therefore in the high-volume reference laboratory, when the majority of blood samples are normal human blood, the H6000 makes it possible to increase precision and accuracy significantly and shorten throughput time while reducing the number of haematologists needed to do the work.

### 22.1.5: H-6000–a unique haematology analyser

*By Jean Pierre Ganon, 39 Boulevard de la Muette, 95140 Garges-les-Gonesse, France*

The H6000 provides a complete haematology profile from a blood sample. The 12 CBC parameters with platelet count, RBC size histogram (Price Jones curve), platelet size histogram along with the XY WBC distribution pattern are reported on one form. With the autoslide option a Wright stained smear is also provided with the results. The design and technical features of the instrument are described and its application to medicine is discussed.

### 22.1.6: The evaluation of two years' experience with Technicon's Hemalog 8 and Hemalog D

*By A. Lučić, R. Grujić, K. Berić, Z. Radujkov, D. Kerac, L. Manić, D. Divjak and K. Antić, Institute of Pathological Physiology and Laboratory Diagnostics, Medical Faculty, Novi Sad, Hajduk Veljkova 1, Yugoslavia*

Basic haematological profiles, including differential leukocyte counting, have been compiled for a group of 25 000 persons with the automated systems—Hemalog 8 and Hemalog D. In the pathological reliability the haematological parameters obtained, have been evaluated and compared with results obtained by manual methods. The authors have demonstrated a positive correlation between results obtained by automated systems and those from the manual technique. Useful data on patients with blood malignant diseases were obtained with the Hemalog D.

### Session 22.2: Heamatology—General

### 22.2.1: Correlation of the HPX and the total white count by optic microscopy

*By B. Cuesta, E. Rocha, J. A. Páramo, A. López Fernandez, R. Sefrés Cordero and J. Fernandez, Clínica Universitaria, Facultad de Medicina de la Univerida de Navarra, Pamplona, Spain*

1000 blood samples analysed with a Technicon Hemalog-D and by conventional microscopy were compared. Correlations between the HPX values with data given by the Hemalog-D were established for (*a*) Neutrophils; (*b*) Lymphocytes; (*c*) LUC (large unstained cells); (*d*) remainder; (*e*) total white cell count. And correlations between the HPX values obtained by conventional microscopy for (1) number of granulocytes (neutrophils, bands, immature granulocytes); (2) number of neutrophils; (3) number of bands; (4) immature granulocytes (HPX) were found. The average and standard deviations of HPX were obtained for those individuals who are out of the two standard deviations, with their neutrophil count (given by the Hemalog-D) and the number of granulocytes obtained by microscopy.

**22.2.2: Automated cytochemistry in the diagnosis of several haematological disorders**

*By M. Fernández de Castro, A. Viloria, M. C. Jiménez Herráez, C. Larrocha, C. Alonso, J. L. Fernández-Chacón and J. M. Gómez Mantilla, Departmento de Laboratorio, C.S.S.S. 'La Paz', Madrid, Spain*

Corresponding features from diverse haematological disorders, obtained from a study on automated cytochemistry, are described. The Hemalog D system was used for the analyses; this provides a differential count of the diverse groups of blood leucocytes, based on their cytochemical behaviour. Different types of leukaemia, both acute and chronic, have been assayed, establishing the quantitative measure of leukaemic and non-leukaemic cells with greater accuracy and precision than is possible with the manual differential. Different types of lymphoproliferative and myeloproliferative diseases were studied, as well as infectious mononucleosis, Hodgkin's disease, myeloma and other disorders like myeloperoxidase deficiency, erythroblastosis and the eosinophilia due to intoxication by toxic colza oil. Characteristic features in the Hemalog D results, each one corresponding to a pathological entity were obtained in all cases. The automated cytochemical method is a good system for detection of several haematological disorders.

**22.2.3: Hereditary myeloperoxidase deficiency: study of 12 cases**

*By M. C. Jiménez Herráez, A. Viloria, M. Fernández de Castro, C. Larrocha, J. L. Fernández-Chacón, M. Salinas and J. M. Gómez Mantilla, Departamento de Laboratorio, C.S.S.S. 'La Paz', Madrid, Spain*

This report concerns 12 cases of hereditary myeloperoxidase (MPO) deficiency. MPO deficiency in the azurophil granulations of the neutrophils and monocytes was detected by an automated cytochemistry system (the Hemalog D system). Cytochemical stainings included peroxidase, Sudan Black B, chloroacetate esterase and alkaline phosphatase. Quantitative enzymatic assay of peroxidase, beta-glucuronidase, acid phosphatase, lysozyme and alkaline phosphatase, were performed, as well as the granulocytic function and electron microscopical study. Five different families were studied, including two generations in each family. Automated cytochemical methods are considered the best way to detect myeloperoxidase deficiency.

**22.2.4: Pseudothrombocytopenia (PTC) produced by platelet agglutinins in EDTA-treated blood**

*By N. Gómez Gómez, M. Lozano Alvarez, F. Olmeda, F. Gómez Reino, M. J. Fernández-Villalta and J. M. Fernández-Rañada, Gran Hospital del Estado, Diego de León, 62, Madrid, Spain*

The authors observed a PTC in EDTA-treated blood from three patients whose platelet counts were obtained with Coulter S Plus counters. The smears showed a normal platelet number and the presence of large-size platelet aggregates. The platelet volume distribution curve in blood treated with EDTA at standard or low concentrations showed the typical flattening observed in platelet anysocitosis. This result was confirmed by the platelet DW. The mean platelet volume obtained with Coulter S Plus Counter was higher than anticoagulants. The addition of platelet-free plasma from these patients to a nor-PRP produced a PTC following incubation at 4°C and 22°C, but not when incubated at 37°C. Tests with neutralizing antibodies and denaturalization with 2-Dithiotreitol suggest that the plasma factor responsible for this phenomenon is an IgM type antibody. Given the high incidence of this phenomenon it is necessary to confirm all thrombocytopenias by a second procedure (chamber or Fonio's) to avoid erroneous diagnosis and subsequent treatment of possible Werlhof's disease.

**22.2.5: A comparison of automatic and manual blood-group antibody screening in blood donors for routine testing**

*By R. L. McShine, P. C. Das, C. Th. Smit Sibinga, Regional Red Cross Blood Bank Gronigen-Drenthe, Groningen, The Netherlands*

The Regional Red Cross Blood Bank in Groningen serves a population of 1·2 M and 13 hospitals, drawing about 56 000 donations annually. Therefore, automation of routine screening tests like ABO and Rhesus grouping, hepatitis and syphilis serology, and screening for irregular blood-group antibodies is of importance for running the laboratory. Preliminary studies have shown that when two types of channels are combined (low ionic strength polybrene and bromelin methyl cellulose), most of the clinically important blood-group antibodies can be detected, but most of these studies were performed on samples from patients. This paper describes how the channels (Marsh and Lâlezari) were built for antibody screening in donor blood. The authors compare auto-analyser results with manual techniques for saline RT, albumin 37°C, indirect antiglobulin test, enzyme 37°C and the low ionic strength method. In January 1981 routine antibody screening was changed from manual testing to automatic screening with an autoanalyser.

**22.2.6: Hemalog D and Hemalog 8 application in peritonitis (P) diagnosis and treatment on continuous ambulatory peritoneal dialysis (CAPD)**

*By R. Selgas, M. Fernandez de Castro, A. Rodriguez Carmona, P. Gomez, J. M. Beberide, O. Ortega and L. Sanchez Sicilia, C.S. La Paz, Madrid, Spain*

Recurrent P has been found to be the most frequent complication with CAPD; under these circumstances the number of leucocytes in the peritoneum increases. A count of leucocytes is very useful for CAPD diagnosis and follow-up. 24 patients on CAPD have passed 69 episodes of P; white cells count out of P in 154 samples (Hemalog-8) was: $276 \pm 36$ $(\bar{x} \pm \text{SEM})/\text{mm}^3$.

*Results:* Leucocyte count in $\text{P/mm}^3$ was $3461 \pm 460$ ($r = 400 - 18800$) and differential count (Hemalog-D): neutrophil: $88 \pm 6\%$ ($\bar{x} \pm$ S.D.); Linphoc: $6{\cdot}5 \pm 6$; Monoc: $3{\cdot}4 \pm 3$; Eosin: $1 \pm 0{\cdot}8$. No significant differences between types of P (according to culture) have been found; eosinophilia is not higher in negative culture P. There is no correlation between cell counts and delay in sampling or symptoms ($r = 0{\cdot}12$). Previous P, sex and age do not correlate with count. Counts in the first six months are slightly higher ($p = 0{\cdot}05$) but neutrophil is no different. The results of cultures have only a small influence on the count. Intra-peritoneal antibiotics provoke a progressive count decrease: ($\bar{x} \pm$ SEM) two–three days $846 \pm 189/\text{mm}^3$; six to eight days $200 \pm 14$; 10–12 days 155–9·4. Evolution is different in those P which have a non-effective treatment: two to three days $1685 \pm 708$; six–eight days $1171 \pm 463$ ($p = 0{\cdot}05$) and 10–12 days $933 \pm 213$ ($p = 0{\cdot}01$). Finally, some cases with a good initial response have been followed by an increased count. The use of the automatic counter is a significant aid for diagnosis and follow-up of peritonitis on CAPD.

**22.2.7: Usefulness of EDTA-blood as samples or controls for haematology tests**

*By I. M. Penttilä, E. Puhakainen and T. Rantanen, Department of Clinical Chemistry, Kuopio University Central Hospital, SF 70210 Kuopio 21, Finland*

A Coulter Counter S-Plus analyser (Coulter Electronics Ltd, Harpenden,

UK) was used for one and a half years for the measurement of parameters in whole blood. During the first months the survival times of blood cells in EDTA-blood were measured—all cell values were found to be constant for up to 6 h at room temperature. Some difficulties with this time have been experienced because health centres are often remote. The second reason for this study was a need for quality control information. Samples analysed daily were stored at +4°C until the following morning, when they were reanalysed for the most important parameters with the Coulter Counter S-Plus analyser. Table 1 presents the results.

*Table 1. Effect of the storage on parameters of EDTA-blood samples (N =950). Mean 1 is for initial values and mean 2 for results after 18 h storage at +4°C. P-values are calculated by the paired t-test for analyses.*

| | WBC 10E9/L | RBC 10E12/L | Hgb g/l |
|---|---|---|---|
| Mean 1 | 8·44 | 4·46 | 133·2 |
| Mean 2 | 8·66 | 4·47 | 133·2 |
| $p<$ | 0·001 | | |

| MCV fl | RDW | PLT 10E9/L | MPV fl | PDW |
|---|---|---|---|---|
| 88·6 | 10·1 | 252·6 | 8·5 | 9·6 |
| 88·1 | 9·6 | 231·9 | 9·1 | 9·5 |
| 0·01 | 0·001 | 0·001 | 0·001 | 0·01 |

Although most differences are signficant, it is evident that in routine haematology practice the daily samples can be used for the control of the Coulter Counter S-Plus analyser, in addition to commercial preparations. EDTA-samples can be analysed the morning after sample taking.

### 22.2.8: Haemoglobin levels of patients attending an under-fives clinic in Benin City, Nigeria

*By O. N. Ogbeide and O. Ogbeide, Department of Chemistry, University of Benin, Benin City, Nigeria*

The importance of anaemia is well known, but has undoubtedly been underestimated. The high incidence of this disease in infancy, especially in developing countries, warrants routine laboratory diagnosis. This is essential for appropriate clinical management, improved surveillance and effective health control. In order to assess the effect of some childhood diseases on haemoglobin values, all patients attending an under-fives clinic in Benin, Nigeria were examined and their Hb levels measured. Such laboratory information using a single haemoglobinometer is presented and the usefulness of this type of routine test is discussed.

### Session 22.3: Electronic Data Processing

### 22.3.1: Real time control procedures and data sample random management using a minicomputer on-line to automatic analysers

*By G. Barbaresi, M. L. Gozzo, G. E. Martorana and C. Zuppi, Clinical Chemistry Laboratory, Policlinico A. Gemelli, Institute of Biological Chemistry, Università Cattolica del Sacro Cuore, Rome, Italy*

The automatic analysers in a clinical chemistry laboratory will normally take care of the analytical work-load. At the same time they usually require a duplication of management procedures. Modern computerized instruments are not able to communicate directly with the central data-bank and do not fit into manual or automatic clerical procedures, which depend upon traditional worksheets. Moreover, security controls must match the speed of the analytical output and follow up instrument performance without delay. For this reason a local information system was developed using an Olivetti P6060 minicomputer on-line to the analysers and exchanging information with the Hospital Data Processing Center (HPDC) by means of floppy discs. An automatic pattern recognition system (optical reader, OPR) performs positive identification of samples which are randomly analysed. Patient number sequence is automatically linked to analytical output. Identified or 'personalized' result records thus obtained are then matched with request records, loaded on a floppy disc from the HDPC which processed the test requests from clinical wards and the patient personal data present in the bank. Thus, a complete clinical report is automatically assembled for each patient, it can be immediately printed in the laboratory and transferred to the data-bank for storing. Moreover, in order to relieve the technical staff from continuous surveillance of analytical output quality, the programs are built to provide alarms of troubles detected by the instrument's own software and to perform additional data validation tests. These latter control result consistency with mathematical comparisons between chemical parameters (discrepancy tests) and with confidence limits suggested by clinical experience (alert tests). Concurrently, a real-time quality-control procedure supplies a statistical index similar to the 'standardized normal deviate' calculated over reference sera results.

An efficient and easy monitoring of the chemical procedures and good result reliability is achieved. Results from different automatic analysers can also be collected, processed, controlled and reported.

### 22.3.2: Specimen identification in the EDP-supported chemical central laboratory

*By H. J. Gibitz and D. Hauch, Zentrallaboratorium der Landeskrankenanstalten, A 5020 Salzburg, Austria*

Mark-sense forms are used in the Zentrallaboratorium requests, the forms contain the name of the patient, a machine-readable patient number with seven digits is applied by the nurse with the help of an ADREMA card. Each mark-sense form is preprinted with one letter and a four-digit specimen number which is machine readable. This specimen identification is also preprinted on six self-adhesive, differently coloured labels on the right margin of the form but is only normally readable. These labels are stuck on to the specimen tubes by the nurse. In the specimen receiving office the tubes are distributed amongst laboratory departments following the lead colours of the labels. The mark-sense forms are read by an IBM 370. The printed working files contain the specimen identifications in an ascendant order (letters and numbers) as well as the corresponding names and numbers of the patients.

The advantages of this system are that the specimen, sample and patient identification is given, without change, from specimen collection to the printing of the patient report. No additional number, a day number for example, is necessary. And an expensive and time-consuming central sample distribution, which is susceptible to the risk of confusion, is not required. Every functional section of the laboratory gets the exactly identified specimen directly from the patient without a time delay.

Details about the production and the preprinting of the mark-sense forms and experiences of this system over three years are presented.

### 22.3.3: Are patient and biological sample identifications compatible?

*By P. Valdiguié, Laboratoire de Biochimie, C. H. U. de Rangueil, 31054 Toulouse Cedex, France*

Correct identification of the many samples handled in different containers within a clinical laboratory is obviously necessary, but it is difficult. This identification must be adapted to the general procedures already in use in the laboratory and must be easily readable by automated machines which are popular. The 'positive' identification systems which are available are not very common yet because they are installed mainly on large and expensive analysers and they do not fit in with the normal organization of the laboratory. Moreover, they use small numbers. Patient identification, on the other hand, usually needs multidigit numbers which are difficult to handle directly during the biological process in the laboratory. The connection between these two incompatible identifications is discussed. A scheme which will be implemented in a large university hospital in Toulouse is reported.

**22.3.4: Bar-code label reading of blood samples from donors simultaneously with blood-grouping in an autoanalyser, for the prevention of clerical errors in a routine blood-bank laboratory**

*By R. L. McShine, A. G. J. de Smit, Z. Smit and C. Th. Smit Sibinga, Regional Red Cross Blood Bank Groningen-Drenthe, Groningen, The Netherlands*

The Regional Red Cross Blood Bank is a large and comprehensive blood centre in the northern Netherlands, which serves 13 hospitals and receives 56 000 donations a year. Automation has been organized to prevent clerical errors in identification and reading of samples, and to increase the standard and accuracy of routine quality control. ABO/Rhesus grouping of donor blood has been automated using a Technicon BG-15 and a connected self-designed autoanalyser with a Marsh and Lalezari channel for automated irregular antibody detection. The sampler has been adapted with a moving white-light source reading device for bar-code identification labels (Codabar). Results are read and decoded in a data-processor, stored on discette and printed for immediate control. The daily results, on discette, are presented to the computer department for updating the donor file and machine-readable quarantine release using a bar-code reader-pen. This procedure has had a significant impact on clerical safety of routine quality control in the laboratory and the quarantine release division of component department.

**22.3.5: Desk-top computer in the clinical laboratory interfaced to automatic multi-channel biochemistry analysers**

*By Yngve Berqqvist and Lars Funding, Department of Clinical Chemistry, Falun Central Hospital, S-791 82 Falun, Sweden, Brendan Henderson, Infax Data H.B., Box 1169, S-163 12 Spanga, Sweden, and Per Eriksson, Scandia Metric AB, Box 1307, S-171 25 Solna, Sweden*

New analytical instruments of increasing sophistication are continuously being introduced into the clinical laboratory. Some drawbacks with these new instruments are inflexible result print-outs, meagre analytical method control reports and the absence of statistics for billing costs. With the help of Scandia Metric AB of Solna, Sweden, two Metric-85 desk top computers were successfully linked to a Greiner G-300 and a Technicon SMA II in order to collect results automatically and to improve performance of the above-mentioned functions. The system hardware consists of two Metric-85 computers each containing a Zilog Z80 CPU, 60 k bytes RAM and two Micropolis mini-floppy disc drives with a storage capacity of 315 k bytes per drive. The hardware system costs less than 70.000 Sw. kr. (12 000 US $). The programs, which are written in BASIC, were developed by Brendan Henderson. The computerized system has the following features: automatic data acquisition; output of patient results on self-adhesive labels; quality control reports for five controls, daily and between days; statistics on the number of analyses ordered by each ward; registration and retrieval of data from disc using patient I.D.; possibility of manual data entry and data editing; data security—passwords inhibit unauthorized access to data. Software and hardware can be used by all laboratory staff after only a few hours' training. This approach demonstrates the use of low-cost desk-top computers for automation in the clinical laboratory.

**22.3.6: Desk-top processor-based interface for chemical analysers**

*By J. I. Dydula, O. S. Lauritsen and T. H. Nielsen, Department of Clinical Chemistry, Frederiksberg Hospital, Copenhagen, Denmark*

Data in a routine laboratory appear in many ways, according to the type of analyser. In the interface area between analyser and computer, small desk-top processors (DTPs) facilitate data handling. Price and flexibility of DTPs allow the decentralized data handling to be tailored to the needs of various workstations. Benefits from this solution are: flexible data capture (on-line/off-line; I.D. numbers/results), quality control and error corrections under the control of the analyst and powerful back-up in case of computer breakdown. This presentation will focus on criteria which could be used when selecting a proper DTP configuration: input/output facilities, programming language, memory size, program library, vendor reliability. The criteria will be illustrated by some experiences with the use of an Apple II.

**22.3.7: On-line data acquisition from a flame photometer**

*By R. A. Lutz, Medical Chemistry Laboratory, Kantonsspital, CH 8401 Winterthur, Switzerland*

The interfacing of a Motorola 6800 microprocessor to a IL-Flame Photometer 343 is described. This microprocessor can store results on an RAM memory module with battery back-up. If the main laboratory minicomputer (NOVA 3) is running and ready, the microprocessor transmits the data, together with the serum number and additional information entered by the laboratory technician. This communication uses the standard RS-232C protocol. If the minicomputer is turned off or is not ready, the microprocessor keeps the results in its own memory until data transfer is possible. This system can be easily modified for other digital laboratory instruments and, therefore, is uniformly applicable. The system has been running for over a year and has proved reliable. Other microprocessors interfaced to other instruments can also be branched to it, simply by adding additional RS-232C interface boards to the microprocessor bus. Only one line to the minicomputer is occupied for up to eight instruments requiring minor software modifications.

**22.3.8: A low-cost data processing system for clinical laboratories**

*By Rafael Clemente, IZASA, S. L., Aragón, 90, Barcelona, Spain*

A low-cost data processing system for clinical laboratories is described, stressing the following functions: information flow in the laboratory; automation of clerical tasks: filing, invoicing, etc.; time distribution study and improvements achieved with the inclusion of the system in a number of laboratories. Expansions of the basic equipment, up to reaching multi-user capability and 'on-line' connection of several automatic analysers, is discussed.

**22.3.9: Rentability and quality advantages—computer and sample distribution in laboratories**

*By Dr Knipps, GFC—Gesellschaft für Computersysteme in der Medizin mbH, Holzhauser Str. 159–165, 1000 Berlin 27, FR Germany*

The GFC-MELAS system has succeeded in gaining a top position in the market during an extremely short period. The system is used in the data processing departments of hospital laboratories.

Only those computers which correspond to most modern technology and flexibility as required for one or more channel measuring instruments are used.

The software is based on modular technique. The high-level programmable language can easily be understood even by personnel who are unfamiliar with data processing. Changes which are necessary in the laboratory, i.e. methods and measuring equipment can easily be performed by the personnel. The PVS Sample Distribution Station 911 is an automatic system which can be connected on-line to laboratory computer systems. The system allows nearly 700 pipettings every hour if one module is used, using three, 2000 pipettings are possible.

Segments can be easily installed during the distribution process. The system saves approximately one to two hours a day by automating the sample-handling stage.

**22.3.10: On-line computers; application in quality control of results**

*By J. M. Navarro and M. J. Alsina, Ambulatorio 'Jaime I', Vilanova i la Geltru, Barcelona, Spain*

Errors that arise in clinical laboratories are produced in three areas: administration, pre-analysis, and the analysis itself. Conventional quality control programmes normally show only the analytical error. Now, by using computers connected on-line to autoanalysers, it is possible to identify a high percentage of all three types of error. The program is divided into the following modules, applied in sequence: analytical quality control; outliers values set by the laboratory according to the type of patient; parameter interrelations; compatibility with diagnosis (abnormal ranges); delta check.

The purpose of this program is to carry out in real time the same operations that would normally be done by the biochemist, leaving him free to deal with those results which do not fit the requirements listed above. A considerable saving in time and greater laboratory efficiency is achieved.

**22.3.11: Integrating a fast computerized analyser (PRISMA) in the organization of a computer-assisted centralized clinical chemical laboratory**

*By C. Pietrzyk, W. Schmaderer, H.-J. Engelhardt, W. Vogt and M. Knedel, Institut für Klinische Chemie, Klinikum Großhadern, Ludwig-Maximilians-Universität München, D-8000 München 70, FR Germany*

A fast multichannel analyser in a clinical laboratory should supply precise, accurate data, quickly and at low cost. The concept of the analytical system determines precision and accuracy of the measurement, while cost and turnaround time for tests are optimal only when the analytical system is fitted to the organization of the laboratory. Bidirectional communication between analyser and laboratory computer system has been realized in the PRIMULAB system developed by the authors. The system offers the following features: requesting analyses and reruns is possible without expending time; patient data do not have to be entered by laboratory personnel; results can be controlled at two levels—analysis-oriented directly on the display unit of the analyser and patient-oriented using the laboratory computer system, that stores more extensive information; long-term storage of quality control data is possible in the laboratory computer.

The laboratory organization must take into account partial or total failure of the multichannel analyser, so that other analysers must replace it. Samples have to be divided and distributed according to the current need, this can be controlled directly by the computer.

**22.3.12: Evaluation of a computer system in a private laboratory**

*By F. Echevarne and J. I. Hornos, Laboratorio de Análisis del Dr F. Echevarne, Barcelona, Spain*

This study investigated the main features that a computer should offer to fit into a private laboratory, the authors discuss features which are absolutely necessary and those which are of marginal importance. The economic advantages of having a computer in a private laboratory are also presented.

**22.3.13: Demands and limitations of the on-line connections between laboratory analysers and computers**

*By V. Seco Torrecillas and J. Aznar Lucea, Laboratorio de Análisis Clínicos; C.S.S.S. 'La Fe', Valencia, Spain*

The authors describe the problems they have found when connecting a computer on-line to their automatic equipment. The features a computer should have are discussed together with the type of equipment that should be connected. They also report on the way data is processed—from the analyser sending it to the computer to the definitive print-out of results. These points are related to the laboratory's work structure and costs and benefits of computerization are given.

**22.3.14: Experiences of a turn-key laboratory computer system**

*By Stanley Wilson, North Manchester General Hospital, Manchester, UK*

The author analyses his experience with a turn-key system in a laboratory of biochemistry in a university hospital over a period of one and a half years, since its installation. The advantages and disadvantages of an on-line connection of a Technicon SMAC, as well as the expansion of the system to the haematology laboratory, are emphasized.

**22.3.15: Six years' experience with an on-line data-base in haemostaseology**

*By Th. Gergely, Institute for Clinical Chemistry and Laboratory Diagnosis of the University of Vienna, Ch. Korninger, 1st Medical Department of the University Hospital of Vienna, and W. Dorda, Institute for Computer Medicine at the University of Vienna, Austria*

The department of haemostaseology of the 1st Medical University Hospital is unique in Vienna, advising other hospitals and supervising the sensitive control of inborn and acquired haemostaseological disorders. To improve efficiency and precision of patient management and control, the traditional database was computerized and linked with the university hospital information system: WAMIS. DOKU is a subsystem of WAMIS and handles the documentation of medically relevant information, such as anamnesia, physical examination and results of diagnostic tests. During the last six years, extensive data on the coagulation system of more than 8000 people have been collected. These data are accessible on-line for routine patient management, such as working schedules for nurses, drug prescriptions, medical reports for referring physicians as well as reminders to physicians and/or patients about necessary control check-ups and required changes in therapy. In addition, a variety of standard and sophisticated mathematical programs are available for on-line scientific work. On-line data processing has proven to be a useful tool to educate personnel—from physicians to nurses, to decrease the paper-work load and to facilitate the use of patient data for scientific and teaching purposes.

### Session 22.4: Cost Analysis

**22.4.1: Economic and quality evaluation obtained with the automation of five immunologic parameters. Experience of six years and 85 000 determinations**

*By F. Malagon, F. L. Elorza, M. E. Dorado and J. M. Corralejo, Departamento Bioquimica Clinica, Hospital Universitario de Sevilla, Spain*

The authors present their experiences, accumulated over six years, on the progressive automation of a laboratory of immunology in a hospital with 1000 beds and 1000 out-patient visits per day. The results obtained with the first automated parameters (IgA, IgG, IgM, $C_3$ and $C_4$) are evaluated; and the technological applications, automated or not, which they set up during the same period of time and with the same personnel are reported. The results are calculated on the basis of 85 000 determinations during the six years of the study, taking into account the evolution undergone by the various parameters (cost, time, etc.) from year to year, with the object of forseeing future needs. The results of automation have been extraordinarily positive, the savings during this time being 16 M pesetas.

**22.4.2: Doubled efficiency by modern analysers**

*By R. Asper, J. W. S. Flückiger and D. J. Vonderschmitt, Central Laboratory of Clinical Chemistry, University Hospital, CH 8091 Zürich, Switzerland*

In university hospital with 1300 beds, automation was brought up to date by replacing 10 batch, or discrete, analysers with five selective or continuous flow analysers. With half of the staff, eight laboratory technicians instead of 16, efficiency was doubled; the average lag between sample arrival and result delivery decreased from three to one and a half hours. Also the emergency results,

25% of the total, are accomplished faster: 90% of these results are delivered in less than one hour. The types of instrument and new organization are discussed.

**22.4.3: Contribution to the setting up, cost analysis and management of a clinical laboratory**

*By F. Lema and R. Alvarez, Laboratorio de Bioquímica, R. S. 'Almirante Vierna', Vigo, Spain*

The authors discuss the steps which need consideration in the setting up of a clinical laboratory. A plan is presented which covers the following points: (1) short-, medium- and long-term objectives. (2) Necessary structures, with reference to: internal structure, personnel, instrumentation, storage, etc. (3) Organization and planning: plans, ways of communication, work, making use of resources, etc. (4) Study of expenses: permanent expenses, variable expenses, expected invoices, starting point, income. (5) Once the laboratory is functioning, an inventory balance of its goods should be made. (6) A study should be made on the efficiency of the personnel. (7) Determine the economic course of the laboratory, by consecutive analysis by ratios of the balance results, permanent active, debts and financial autonomy, in comparison with the expected economic objectives.

**20.2.20: Evaluation of an autoanalyser for determination of glucose by immobilized enzymes**

*By J. Mateo and S. Valle, Department of Clinical Chemistry, University Hospital, Sevilla, Spain*

The aim of this presentation is to publish the results obtained in an evaluation of an autoanalyser (Model C-202) for the determination of glucose by immobilized enzymes (glucose-oxidase). The protocol described by Broughton *et al.* (*Annals of Clinical Biochemistry*, **11** [1974], 207–218) was followed for the following parameters: precision at three concentrations over 20 days; accuracy by means of primary standards; linearity at various concentrations from 0 to 400 mg%; carry-over and drift. Similarly, cost per sample, response time and correlation with other enzymatic methods was studied. Although the results obtained show that the values are satisfactory, the small sample volume (2 μl) may introduce errors necessitating some precautions.

*Editor's note: this abstract is not in sequence because it arrived late.*

### Notes for Authors

*Journal of Automatic Chemistry* covers all aspects of automation and mechanization in analytical, clinical and industrial environments. The *Journal* publishes original research papers; short communications on innovations, techniques and instrumentation, or current research in progress; reports on recent commercial developments; and meeting reports, book reviews and information on forthcoming events. All research papers are refereed.

### Manuscripts

Two copies of articles should be submitted to the Editor. All articles should be typed in double spacing with ample margins, on one side of the paper only. The following items should be sent: (1) a title-page including a brief and informative title, avoiding the word 'new' and its synonyms; a full list of authors with their affiliations and full addresses; (2) an abstract of about 250 words—this should succinctly describe the scope of the contribution and highlight significant findings or innovations; it should be written in a style which can easily be translated into French and German; (3) the main text with sections and subsections numbered; (4) appendices (if any); (5) references; (6) tables, each table on a separate sheet and accompanied by a caption; (7) illustrations (diagrams, drawings and photographs) numbered in a single sequence from 1 upwards and with the author's name on the back of every illustration; captions to illustrations should be typed on a separate sheet.

### References

References should be indicated in the text by numbers following the author's name, i.e. Skeggs [6]. In the reference section they should be arranged thus:

to a journal

Manka, D. P., *Journal of Automatic Chemistry*, **3** (1981), 119.

to a book

Malmstadt, H. V., in *Topics in Automatic Chemistry*, Ed. Stockwell, P. B. and Foreman, J. K. (Horwood, Chichester, 1978), p. 68.

### Illustrations

Line diagrams are preferred to photographs. Original copies of diagrams and drawings should be supplied, and should be drawn to be suitable for reduction to the page or column width of the *Journal*, i.e. to 85 mm or 179 mm, with special attention to lettering size. Photographs may be sent as glossy prints or as negatives.

### Proofs and offprints

The principal or corresponding author will be sent galley proofs for checking and will receive 50 offprints free of charge. Additional offprints may be ordered on a form which accompanies the proofs.

*Manuscripts should be sent to the Editor: Dr Peter B. Stockwell, Plasma-Therm Ltd, 6 Station Road, London SE20 7BQ.*

